# Large-order NSPT for lattice gauge theories with fermions: the plaquette in massless QCD

**DOI:** 10.1140/epjc/s10052-018-6458-9

**Published:** 2018-11-24

**Authors:** L. Del Debbio, F. Di Renzo, G. Filaci

**Affiliations:** 1 Higgs Centre for Theoretical Physics, School of Physics & Astronomy, University of Edinburgh, Edinburgh, EH9 3FD UK; 2 Dipartimento di Scienze Matematiche, Fisiche e Informatiche, Università di Parma and INFN, Gruppo Collegato di Parma, 43100 Parma, Italy

## Abstract

Numerical Stochastic Perturbation Theory (NSPT) allows for perturbative computations in quantum field theory. We present an implementation of NSPT that yields results for high orders in the perturbative expansion of lattice gauge theories coupled to fermions. The zero-momentum mode is removed by imposing twisted boundary conditions; in turn, twisted boundary conditions require us to introduce a smell degree of freedom in order to include fermions in the fundamental representation. As a first application, we compute the critical mass of two flavours of Wilson fermions up to order $O(\beta ^{-7})$ in a ${{\mathrm{{\mathrm {SU}}}}}(3)$ gauge theory. We also implement, for the first time, staggered fermions in NSPT. The residual chiral symmetry of staggered fermions protects the theory from an additive mass renormalisation. We compute the perturbative expansion of the plaquette with two flavours of massless staggered fermions up to order $O(\beta ^{-35})$ in a ${{\mathrm{{\mathrm {SU}}}}}(3)$ gauge theory, and investigate the renormalon behaviour of such series. We are able to subtract the power divergence in the Operator Product Expansion (OPE) for the plaquette and estimate the gluon condensate in massless QCD. Our results confirm that NSPT provides a viable way to probe systematically the asymptotic behaviour of perturbative series in QCD and, eventually, gauge theories with fermions in higher representations.

## Introduction

The success of perturbation theory in High Energy Physics (HEP) can hardly be denied. In particular, in asymptotically free theories, field correlators at short distances are reliably approximated by perturbative expansions in the running coupling at a large momentum scale. At the same time, even in these (lucky) cases, it is mandatory to have some control on nonperturbative effects, i.e. contributions that scale like powers of the QCD scale $\Lambda _\mathrm {QCD}$. We will often refer to these as *power corrections*. A tool to take the latter into account was suggested back in the late seventies. This goes under the name of QCD sum rules, or Shifman-Vainshtein-Zakharov (SVZ) sum rules [1, 2]. One of the authors defined the method as “an expansion of the correlation functions in the vacuum condensates” [3]. These condensates are the vacuum expectation value of the operators that emerge in the Operator Product Expansion (OPE) for the relevant correlation function. In the OPE formalism the condensates are fundamental quantities, which are in principle supposed to parametrise power corrections in a universal way. By determining the value of a condensate in one context, one gains insight into different physical processes; this has in turn motivated several approaches to the determination of condensates. Having said all this, the sad news is that not all the condensates have actually the same status. In particular not all the condensates can be defined in a neat way, which ultimately means disentangled from perturbation theory. While this is the case for the chiral condensate, the same cannot be said for the gluon condensate, which is the one we will be concerned with in this work.

Based on a separation of scales, the OPE makes pretty clear what can/must be computed in perturbation theory, i.e. the Wilson coefficients. Still, this does not automatically imply that perturbative and nonperturbative contributions are separated in a clear-cut way. The key issue is that perturbative expansions in HEP are expected to be asymptotic ones on very general grounds. In particular, the series in asymptotically free theories are plagued by ambiguities which are due to so-called infrared renormalons [4, 5]. From a technical point of view, renormalons show up as singularities which are encountered if one tries to Borel resum the perturbative series. All in all, there is a power-like ambiguity in any procedure one can devise in order to sum the series, and this ambiguity unavoidably reshuffles perturbative and nonperturbative contributions in the structure of the OPE. Being the Wilson coefficients affected by ambiguities that are power corrections, the general strategy is to reabsorb the latter in the definition of the condensates. This amounts to a prescription to give a precise meaning both to the perturbative series and to the condensates that appear in the OPE.

The idea of determining the gluon condensate from nonperturbative (Monte Carlo) measurements in lattice gauge theories dates back to the eighties and early nineties [6–9]. Based on symmetry grounds and dimensional counting, the two leading contributions in the OPE for the basic plaquette are given by the identity operator and the gluon condensate. Both operators appear multiplied by Wilson coefficients that can be computed in perturbation theory, and in particular the coefficient that multiplies the identity operator is simply the perturbative expansion of the plaquette. Other operators that appear in the OPE are of higher dimension, and their contributions are therefore suppressed by powers of $a \Lambda _\mathrm {QCD}$. Subtracting from a nonperturbative (Monte Carlo) measurement of the plaquette the sum of the perturbative series, and repeating the procedure at different values of the coupling, the signature of asymptotic scaling, i.e. the signature of a quantity of (mass) dimension four, should become visible. With renormalons attracting more and more attention, it eventually became clear that such a procedure must be deeply affected by the ambiguities we discussed above, suggesting that a precise definition of the resummed perturbative expansion is necessary.

In the meantime Numerical Stochastic Perturbation Theory (NSPT) [10] was developed as a new tool for computing high orders in lattice perturbation theory. NSPT paved the way to the evaluation of many more terms in the perturbative expansion of the plaquette, and in turn made it at least conceivable that the behaviour of the series could be understood at the level of pinning down the correct order of magnitude of the ambiguity involved. Results of early investigations [11] were interesting: for the first time, it was clear that very high order contributions can be computed in perturbative series for lattice gauge theories. Unfortunately the pioneering NSPT studies of that time were far away from computing the series up to the orders at which the renormalon growth actually shows up in its full glory. With limited computing power available, a way out was sought in the form of a change of scheme (i.e. a scheme in which the renormalon behaviour is best recognised, possibly at lower orders than in the lattice scheme). Still, the numerical results were in the end puzzling as for consequences, since trying to sum the series from the information available even suggested the idea that an unexpected contribution from a dimension-2 operator was present [12]. Other attempts were made [13], but it eventually took roughly twenty years before the renormalon behaviour was actually captured [14–17], needless to say, via NSPT.^1^ In ${{\mathrm{{\mathrm {SU}}}}}(3)$ Yang–Mills theory the IR renormalon was indeed directly inspected, and the finite-size effects that are unavoidable on finite lattices assessed. The bottom line is that the victory is twofold. On one side, the renormalon growth is indeed proved to be present as conjectured (ironically, in a scheme – the lattice – which one would have regarded as the very worst to perform the computations). Given this, one has a prescription to sum the series and perform the subtraction (if sufficiently high orders are available, one can look for the inversion point in the series, where contributions start to grow and a minimum indetermination in summing the series can be attained).

The present work is a first attempt at performing the determination of the gluon condensate from the plaquette in full QCD, i.e. with fermionic contributions taken into account. The main focus here is in developing the NSPT technology, and present a first set of results, which allow a definition of the gluon condensate. In particular for the first exploration, we use existing Monte Carlo simulations for the plaquette in full QCD, as detailed below. Having ascertained that the procedure is viable, a precise determination of the condensate in full QCD will require a dedicated Monte Carlo simulation, with a careful choice of the fermionic action. On top of being interesting *per se*, the methodology presented here opens the way to other applications, in which different colour groups and different matter contents can be investigated. The final goal would be to inspect whether in a theory that has an IR fixed point, the renormalon growth is tamed, as one would expect in theories where the condensates vanish. We defer these questions to future investigations, hoping to gain extra insight into the task of identifying the boundaries of the conformal window.

The paper is organised as follows. In Sect. 2 we review briefly how NSPT can be applied to lattice gauge theories. In Sect. 3 twisted boundary conditions for fermions in the fundamental representation are introduced. In Sect. 4 we discuss how to take into account fermions with smell in NSPT. We present our results for the expansion of the critical mass of Wilson fermions in Sect. 5, and for the expansion of the plaquette with staggered fermions in Sect. 6. In Sect. 7 we investigate the asymptotic behaviour of the expansion of the plaquette and extract the gluon condensate in massless QCD. In Sect. 8 we draw our conclusions and present some possible future steps.

## Lattice gauge theories in NSPT

Let us here summarise the main steps in defining NSPT for lattice gauge theories. Rather than trying to give a comprehensive review of the method, we aim here to introduce a consistent notation that will allow us to discuss the new developments in the rest of the paper. For a more detailed discussion of the NSPT formulation, the interested reader can consult e.g. Ref. [18], whose notation we shall try to follow consistently.^2^ In particular, we assume to work with a hypercubic lattice with volume $L^4=a^4N^4$ and assume the lattice spacing *a* to be 1, unless where stated otherwise. We use *x*, *y*, *z* for position indices, $\mu ,\nu ,\rho =1,\ldots ,4$ for Lorentz indices and $\alpha ,\beta ,\gamma =1,\ldots ,4$ for Dirac indices.

The original formulation of NSPT is based on the Stochastic Quantization formulation of lattice field theories, in the case at hand lattice gauge theories. For the purposes of this study, we focus on gauge theories that are defined by the Euclidean Wilson action for the gauge group ${{\mathrm{{\mathrm {SU}}}}}(N_c)$:

1 $$\begin{aligned} S_G\left[ U\right] = -\frac{\beta }{2 N_c} \sum _\Box \mathrm {Tr} \left( U_\Box + {U_\Box }^\dagger \right) \, , \end{aligned}$$

where $U_\Box$ is the product of the link variables, denoted $U_{\mu }(x)$, around the $1\times 1$ plaquette $\Box$, and the sum extends over all the plaquettes in the lattice. Introducing a stochastic time *t*, a field $U_{\mu }(x;t)$ can be defined that satisfies the Langevin equation

2 $$\begin{aligned} \frac{\partial }{\partial t} U_{\mu }(x;t) = i \Big [ -\nabla _{x\mu } S_G[U_{\mu }(x;t)] - \eta _{\mu }(x;t) \Big ] U_{\mu }(x;t)\, .\nonumber \\ \end{aligned}$$

As detailed in Appendix A, we have denoted by $\nabla _{x\mu }$ the left derivative in the group; $\eta$ is a stochastic variable defined in the algebra of the group,

3 $$\begin{aligned} \eta _{\mu }(x;t) = \sum _a T^a \eta _{\mu }^a(x;t) \, , \end{aligned}$$

where $T^a$ are the generators of the group, and $\eta _{\mu }^a(x;t)$ are Gaussian variables such that

4 $$\begin{aligned}&\langle \eta _{\mu }^a(x;t) \rangle = 0 , \nonumber \\&\langle \eta _{\mu }^a(x;t)\, \eta ^{b}_{\nu }(y;t')\rangle = 2 \delta ^{ab} \delta _{\mu \nu } \delta _{xy} \delta (t-t')\, . \end{aligned}$$

The key point of Stochastic Quantization is that the large-*t* distribution of observables built from the solution of the Langevin equation above corresponds to the distribution that defines the path integral of the quantum theory [19, 20]:

5 $$\begin{aligned} \lim _{t\rightarrow \infty } \langle O[U(t)]\rangle = \frac{1}{Z} \int \mathcal {D}U\, e^{-S_G[U]} O[U]\, . \end{aligned}$$

In order to develop NSPT, the dynamical variables $U_{\mu }(x;t)$ can be expanded in powers of the coupling constant *g*, which is given in the lattice formulation by $\beta ^{-1/2}$:

6 $$\begin{aligned} U_{\mu }(x;t) \mapsto 1 + \sum _{k=1} \beta ^{-k/2} U_{\mu }^{(k)}(x;t)\, . \end{aligned}$$

Solving the Langevin equation, Eq. (2), order by order in $\beta ^{-1/2}$ yields a system of coupled equations for the *perturbative components* of the link variables $U_{\mu }^{(k)}(x;t)$.

Expanding the solution of Langevin equation in powers of the coupling is a standard approach to proving the equivalence of stochastic and canonical quantisation, i.e. Eq. (5) [21], and was the starting point for stochastic perturbation theory: with this respect NSPT is just the numerical implementation of the latter on a computer. The idea of studying the convergence properties of a stochastic process order by order after an expansion in the coupling is actually quite general. In this spirit different NSPT schemes can be set up, also based on stochastic differential equations different from Langevin [22, 23].

**Euler integrator** Discretising the stochastic time in steps of size $\epsilon$ allows a numerical integration of the Langevin equation,

7 $$\begin{aligned} U_{\mu }(x;t+\epsilon ) = e^{-F_{\mu }(x;t)}\, U_{\mu }(x;t)\, , \end{aligned}$$

where the force driving the evolution is

8 $$\begin{aligned} F_{\mu }(x;t)&= i\left[ \epsilon \nabla _{x\mu } S_G[U(t)] + \sqrt{\epsilon }\, \eta _{\mu }(x;t)\right] \nonumber \\&= \epsilon \, \frac{\beta }{2 N_c} \sum _{U_\Box \supset U_{\mu }(x)} {{\mathrm{\Pi _\mathfrak {g}}}}(U_\Box ) + \sqrt{\epsilon } \,\eta _{\mu }(x;t)\, \end{aligned}$$

and the operator ${{\mathrm{\Pi _\mathfrak {g}}}}$ projects on the algebra (see Appendix A). Note that Eq. (8) does not lend itself to a perturbative solution in powers of $\beta ^{-1/2}$, since there is a mismatch between the deterministic drift term, which starts at order $\beta ^{1/2}$, and the stochastic noise, which is of order $\beta ^0$. This is easily resolved by rescaling the integration step by a factor of $\beta$, so that both contributions start at order $\beta ^{-1/2}$. Denoting the new time step $\tau = \epsilon \beta$, the force term becomes

9 $$\begin{aligned} F_{\mu }(x;t) = \frac{\tau }{\beta } \nabla _{x\mu } S_G[U(t)] + \sqrt{\frac{\tau }{\beta }} \, \eta _{\mu }(x;t) \, . \end{aligned}$$

Expanding *F* in powers of $\beta ^{-1/2}$,

10 $$\begin{aligned} F_{\mu }(x;t) = \sum _{k=1} \beta ^{-k/2} F^{(k)}_{\mu }(x;t)\, , \end{aligned}$$

leads to a system of coupled equations for the evolution of the coefficients of the perturbative expansion of *U*. Omitting Lorentz and position indices, we get

11a $$\begin{aligned} U^{(1)}(t+\tau )&= U^{(1)}(t) - F^{(1)}(t) \end{aligned}$$

11b $$\begin{aligned} U^{(2)}(t+\tau )&= U^{(2)}(t) - F^{(2)}(t) + \frac{1}{2} F^{(1)}(t)^2 \nonumber \\&\quad - F^{(1)}(t) U^{(1)}(t) \nonumber \\&\ldots \end{aligned}$$

where $\eta$ only contributes to the $F^{(1)}$ term.

**Stochastic gauge fixing** The zero modes of the gauge action do not generate a deterministic drift term in the Langevin equation, and therefore their evolution in stochastic time is entirely driven by the stochastic noise, which gives rise to diverging fluctuations. This phenomenon is well known since the early days of NSPT, see e.g. Ref. [24], and is cured by the so-called stochastic gauge fixing procedure [25] applied to the theory formulated on the lattice. The procedure implemented in this work alternates an integration step as described above with a gauge transformation:

12 $$\begin{aligned} U_{\mu }(x) \mapsto e^{w(x)} U_{\mu }(x) e^{-w(x+{\hat{\mu }})}\, , \end{aligned}$$

where the field *w*(*x*) is defined in the algebra of the group,

13 $$\begin{aligned} w(x) = - \alpha {{\mathrm{\Pi _\mathfrak {g}}}}\left( \sum _\mu \nabla ^*_\mu U_{\mu }(x)\right) \,. \end{aligned}$$

$\alpha$ is a free parameter, which we choose equal to 0.1 and $\nabla ^*_\mu$ is the backward derivative in direction $\mu$. Note that there is nothing compelling in the choice for *w*(*x*). In this work we make the same choice as in Ref. [24], which is slightly different from the one adopted in Ref. [18]: the corresponding gauge transformation does not lead, if iterated, to the Landau gauge. In NSPT the gauge transformation is expanded in powers of the coupling,

14 $$\begin{aligned} w(x) = \sum _{k=1} \beta ^{-k/2} w^{(k)}(x)\, , \end{aligned}$$

and the transformation in Eq. (12) is implemented order by order in perturbation theory.

The combined step for the integrator adopted in this work can be summarised as

15a $$\begin{aligned} U_{\mu }(x)'&= e^{-F_{\mu }(x;t)}\, U_{\mu }(x;t)\, , \end{aligned}$$

15b $$\begin{aligned} U_{\mu }(x;t+\tau )&= e^{w[U'](x)} U_{\mu }(x)' e^{-w[U'](x+{\hat{\mu }})}\, , \end{aligned}$$

where all the terms are expanded in powers of $\beta ^{-1/2}$, and the perturbative components are updated.

**Runge–Kutta integrator** Higher order integrators, in particular Runge–Kutta schemes, have been used for the lattice version of the Langevin equation since the early days [20]. A new, very effective second-order integration scheme for NSPT in lattice gauge theories has been introduced in Ref. [15]. While we have tested Runge–Kutta schemes ourselves for pure gauge NSPT simulations, in this work we adhere to the simpler Euler scheme: when making use of the (standard) stochastic evaluation of the fermionic equations of motion (see Sect. 4), Runge–Kutta schemes are actually more demanding (extra terms are needed [26, 27]).

## Twisted boundary conditions and smell

When a theory is defined in finite volume, the fields can be required to satisfy any boundary conditions that are compatible with the symmetries of the action. We adopt twisted boundary conditions (TBC) [28] in order to remove the zero-mode of the gauge field, and have an unambiguous perturbative expansion, which is not plagued by toron vacua [29]. The gauge fields undergo a constant gauge transformation when translated by a multiple of the lattice size; therefore twisted boundary conditions in direction ${\hat{\nu }}$ are

16 $$\begin{aligned} U_\mu (x+L{\hat{\nu }})=\Omega _\nu U_\mu (x)\Omega _\nu ^\dag \,, \end{aligned}$$

where $\Omega _\mu \in {{\mathrm{{\mathrm {SU}}}}}(N_c)$ are a set of constant matrices satisfying

17 $$\begin{aligned} \Omega _\nu \Omega _\mu = z_{\mu \nu } \Omega _\mu \Omega _\nu \,,\qquad z_{\mu \nu }\in Z_{N_c}\,. \end{aligned}$$

Fermions in the adjoint representation can be introduced in a straightforward manner; the boundary conditions with the fermionic field in the matrix representation read

18 $$\begin{aligned} \psi (x+L{\hat{\nu }})=\Omega _\nu \psi (x)\Omega _\nu ^\dag \,. \end{aligned}$$

The inclusion of fermions in the fundamental representation is not straightforward; indeed, the gauge transformation for the fermions when translated by a multiple of the lattice size reads

19 $$\begin{aligned} \psi (x+L{\hat{\nu }})=\Omega _\nu \psi (x)\, , \end{aligned}$$

leading to an ambiguous definition of $\psi (x+L{\hat{\mu }}+L{\hat{\nu }})$. An idea to overcome this problem, proposed in Ref. [30] and implemented e.g. in Ref. [31], is to introduce a new quantum number so that fermions exist in different copies, or *smells*, which transform into each other according to the antifundamental representation of ${{\mathrm{{\mathrm {SU}}}}}(N_c)$. The theory has a new global symmetry, but physical observables are singlets under the smell group. Thus, configurations related by a smell transformations are equivalent, and in finite volume we are free to substitute Eq. (19) with

20 $$\begin{aligned} \psi (x+L{\hat{\nu }})_{ir}=\sum _{j,s}\big (\Omega _\nu \big )_{ij} \psi (x)_{js}\big (\Lambda _\nu ^\dag \big )_{s r}\, , \end{aligned}$$

where $\Lambda _\nu \in {{\mathrm{{\mathrm {SU}}}}}(N_c)$. It is useful to think of the fermion field as a matrix in colour-smell space. If the transformation matrices in smell space satisfy the same relations as in Eq. (17) (in particular we choose them to be equal to the $\Omega$s), then twisted boundary conditions are well-defined.

It is worth pointing out that, through a change of variable in the path integral [32, 33], twisted boundary conditions could be equivalently implemented by multiplying particular sets of plaquettes in the action by suitable elements of $Z_{N_c}$ and considering the fields to be periodic. This change of variable works only in the pure gauge or fermions in the adjoint representation cases. Thus, the explicit transformation of Eq. (20) is required when fermions in the fundamental representation with smell are considered.

## Fermions in NSPT

If $S_F=\sum _{x,y}{\bar{\psi }}(x) M[U] \psi (y)$ is the action of a single fermion, then dynamical fermions in NSPT can be included thanks to a new term in the drift, as shown in Refs. [20, 34]: the determinant arising from $N_f$ degenerate fermions can be rewritten as

21 $$\begin{aligned} \det (M)^{N_f} = \exp \left( N_f{{\mathrm{\mathrm {Tr}}}}\ln M\right) \end{aligned}$$

and can be taken into account by adding $-{N_f}{{\mathrm{\mathrm {Tr}}}}\ln M$ to the gauge action. From the Lie derivative of the additional term and recalling that a rescaled time step $\tau =\epsilon /\beta$ is used in the Euler update, we obtain the new contribution

22 $$\begin{aligned} F^{f}_\mu (x)= -i\,\frac{\tau N_f}{\beta }\sum _a T^a {{\mathrm{\mathrm {Tr}}}}(\nabla ^a_{x\mu } M) M^{-1} \end{aligned}$$

to be added to the pure gauge drift. It is important to note that the coefficient of $iT^a$ is purely real because the Wilson operator is $\gamma _5$-Hermitian and the staggered operator is antihermitian: this is consistent with the drift being an element of the algebra. The trace can be evaluated stochastically: Eq. (22) is replaced by

23 $$\begin{aligned} F^{f}_\mu (x) =-i\frac{\tau N_f}{\beta }\sum _a T^a {{\mathrm{{\text {Re}}}}}\xi ^*(\nabla ^a_{x\mu } M) M^{-1}\xi \end{aligned}$$

thanks to the introduction of a new complex Gaussian noise $\xi$ satisfying^3^

24 $$\begin{aligned} \mathinner {\langle {\xi ^*(y)_{\beta i r}\xi (z)_{\gamma js}}\rangle } = \delta _{yz} \delta _{\beta \gamma }\delta _{ij}\delta _{rs}\,. \end{aligned}$$

The real part must be enforced, otherwise the dynamics would lead the links out of the group since the drift would be guaranteed to be in the algebra only on average. In NSPT, the Dirac operator inherits a formal perturbative expansion from the links, $M=\sum _{n=0}^\infty \beta ^{-n} M^{(n)}$, so the inverse $\psi =M^{-1}\xi$ can be computed efficiently from the knowledge of the inverse free operator via the recursive formula

25a $$\begin{aligned} \psi ^{(0)}&={M^{(0)}}^{-1}\xi \end{aligned}$$

25b $$\begin{aligned} \psi ^{(n)}&=-{M^{(0)}}^{-1}\sum _{j=0}^{n-1}M^{(n-j)}\psi ^{(j)}\,. \end{aligned}$$

The inverse of the free operator is conveniently applied in Fourier space.

If fermions have smell, then the rescaling $N_f\rightarrow N_f/N_c$ is required in order to have $N_f$ flavours in the infinite-volume limit. In other words, this is the same as considering the $N_c$th root of the determinant of the fermion operator. In principle such rooted determinant could come from a nonlocal action, because twisted boundary conditions break the invariance under smell transformations. Nevertheless, this rooting procedure is sound since we know in advance that in the infinite-volume limit all the dependence on boundary conditions will be lost and the determinant will factorise as the fermion determinant of a single smell times the identity in smell space. It is also possible to show with arguments similar to those presented in Ref. [35] that, if the theory without smell is renormalisable, this operation leads to a perturbatively renormalisable theory as well. Below we describe in detail Wilson and staggered fermions in the fundamental representation, so we explicitly rescale $N_f\rightarrow N_f/N_c$. It is also important to remember that the fermion field, seen as a matrix in colour-smell space, is not required to be traceless, thus its Fourier zero-mode does not vanish: we require antiperiodic boundary conditions in time direction not to hit the pole of the free propagator in the massless case. We avoid twisted boundary conditions in time direction because in the massless case it might happen for the free fermion propagator to develop a pole at some particular momenta.

### Wilson fermions

The Wilson Dirac operator and its Lie derivative are

26a $$\begin{aligned} M_{y\beta i r, z\gamma j s} =\,&(m+4)\delta _{rs}\delta _{yz} \delta _{\beta \gamma }\delta _{ij}\nonumber \\&+\sum _\mu \left[ D(\mu )+\gamma _5 D(\mu )^\dag \gamma _5\right] _{y\beta i r, z\gamma j s} \end{aligned}$$

26b $$\begin{aligned} \nabla ^a_{x,\mu }M_{y\beta i r, z\gamma j s} =\,&i\delta _{xy}[T^aD(\mu )]_{y\beta i r, z\gamma j s}\nonumber \\&-i\delta _{xz}[\gamma _5D(\mu )^\dag \gamma _5T^a]_{y\beta i r, z\gamma j s}\,, \end{aligned}$$

where the non-diagonal term has been expressed through

27 $$\begin{aligned} D(\mu )_{y\beta i r, z\gamma j s}=-\frac{1}{2}\delta _{rs}\delta _{y,z-{\hat{\mu }}}(1-\gamma _\mu )_{\beta \gamma }U_\mu (y)_{ij}\,. \end{aligned}$$

We must give a perturbative structure to the mass $m=\sum _{n=0}^\infty \beta ^{-n} m^{(n)}$ to account for an additive mass renormalisation, see Sect. 5. The stochastic evaluation of the trace leads to

28 $$\begin{aligned} \xi ^*(\nabla ^a_{x\mu } M) M^{-1}\xi= & {} i{{\mathrm{\mathrm {Tr}}}}T^a \sum _\beta \left( \varphi ^{(\mu )}(x)_\beta \,\xi (x)^\dag _\beta \right. \nonumber \\&\left. -\psi (x)_\beta \,{\tilde{\varphi }}^{(\mu )}(x)_\beta ^\dag \right) \,, \end{aligned}$$

where $\varphi ^{(\mu )}=D(\mu )\psi$, ${\tilde{\varphi }}^{(\mu )}=\gamma _5D(\mu )\gamma _5\xi$ and the fermion fields have been represented as matrices in colour-smell space. After taking the real part, the fermion drift can be finally written as

29 $$\begin{aligned} F^{f}_\mu (x)_{ij}=&\frac{1}{2}\frac{N_f}{N_c}\frac{\tau }{\beta }\sum _a T^a_{ij} {{\mathrm{\mathrm {Tr}}}}T^a \sum _\beta \left[ \left( \varphi ^{(\mu )}(x)_\beta \,\xi (x)^\dag _\beta \right. \right. \nonumber \\&\left. \left. -\psi (x)_\beta \,{\tilde{\varphi }}^{(\mu )}(x)_\beta ^\dag \right) -\text {h.c.}\right] \nonumber \\ =&\frac{1}{2}\frac{N_f}{N_c}\frac{\tau }{\beta }{{\mathrm{\Pi _\mathfrak {g}}}}\left[ \sum _\beta \left( \varphi ^{(\mu )}(x)_\beta \,\xi (x)^\dag _\beta \right. \right. \nonumber \\&\left. \left. +{\tilde{\varphi }}^{(\mu )}(x)_\beta \,\psi (x)_\beta ^\dag \right) \right] _{ij}\,. \end{aligned}$$

In Appendix B the actual implementation of the fermion drift is described (only one of the two terms in Eq. (29) is actually needed).

With the Fourier transform described in Appendix C, the inverse free Wilson operator with twisted boundary conditions is diagonal in momentum space and can be expressed as

30 $$\begin{aligned} {M^{(0)}}^{-1}_{k,p}=\delta _{k_\parallel p_\parallel }\delta _{k_\perp p_\perp }\frac{2\sum _{\mu }\sin ^2\frac{k_\mu }{2}+m^{(0)}-i\sum _{\mu }\gamma _\mu \sin k_\mu }{\left( 2\sum _{\mu }\sin ^2\frac{k_\mu }{2}+m^{(0)}\right) ^2+\sum _{\mu } \sin ^2 k_\mu }\,.\nonumber \\ \end{aligned}$$

### Staggered fermions

We implemented for the first time staggered fermions in NSPT. The staggered field has no Dirac structure and describes four physical fermions in the continuum limit. Therefore, we rescale $N_f\rightarrow N_f/4$ and the staggered operator is understood to be rooted when the number of flavour is not a multiple of four. The staggered Dirac operator and its Lie derivative are

31a $$\begin{aligned} M_{y i r, z j s} =\,&m\delta _{rs}\delta _{yz} \delta _{ij}+\sum _\mu \left[ D(\mu )-D(\mu )^\dag \right] _{y i r, z j s} \end{aligned}$$

31b $$\begin{aligned} \nabla ^a_{x,\mu }M_{y i r, z j s} =\,&i\delta _{xy}[T^aD(\mu )]_{y i r, z j s}\nonumber \\&+i\delta _{xz}[D(\mu )^\dag T^a]_{y i r, z j s}\,, \end{aligned}$$

where the non-diagonal term has been expressed through

32 $$\begin{aligned} D(\mu )_{y i r, z j s}=\frac{1}{2}\alpha _\mu (y)\delta _{rs}\delta _{y,z-{\hat{\mu }}}U_\mu (y)_{ij} \end{aligned}$$

and $\alpha _\mu (x)=(-1)^{\sum _{\nu =1}^{\mu -1}x_\nu }$ is the staggered phase. The stochastic evaluation of the trace is analogous to the Wilson fermion case and Eq. (28) becomes

33 $$\begin{aligned} \xi ^*(\nabla ^a_{x\mu } M) M^{-1}\xi= & {} i{{\mathrm{\mathrm {Tr}}}}T^a \left( \varphi ^{(\mu )}(x)\,\xi (x)^\dag \right. \nonumber \\&\left. -\psi (x)\,{\tilde{\varphi }}^{(\mu )}(x)^\dag \right) \,, \end{aligned}$$

with $\varphi ^{(\mu )}=D(\mu )\psi$ and ${\tilde{\varphi }}^{(\mu )}=-D(\mu )\xi$, leading to the final expression

34 $$\begin{aligned} F^{f}_\mu (x)_{ij}= & {} \frac{1}{2}\frac{N_f}{4N_c}\frac{\tau }{\beta }{{\mathrm{\Pi _\mathfrak {g}}}}\left( \varphi ^{(\mu )}(x)\,\xi (x)^\dag \right. \nonumber \\&\left. +\,{\tilde{\varphi }}^{(\mu )}(x)\,\psi (x)^\dag \right) _{ij}\,. \end{aligned}$$

Again, the actual implementation of the staggered drift is shown in Appendix B.

With the Fourier transform described in Appendix C, the inverse free staggered operator with twisted boundary conditions is found to be

35 $$\begin{aligned} {M^{(0)}}^{-1}_{k,p}=\delta _{k_\perp p_\perp }\frac{m\delta _{k_\parallel p_\parallel }-i\sum _\mu \sin k_\mu \,{\bar{\delta }}(k_\parallel +\pi {\bar{\mu }}-p_\parallel )}{\sum _\mu \sin ^2 k_\mu +m^2}\,, \end{aligned}$$

where ${\bar{1}}=0$, $\overline{\mu +1}={\bar{\mu }}+{\hat{\mu }}$ and ${\bar{\delta }}$ is the periodic Kronecker delta, with support in $0\mod 2\pi$. The propagator is not diagonal in momentum space because the action depends explicitly on the position through $\alpha _\mu (x)$, but it is simple enough to avoid a complete matrix multiplication over all the degrees of freedom. If we aim to compute ${M^{(0)}}^{-1}v$ for some field *v* in momentum space, it is useful to represent $v(p_\parallel )_{p_\perp }$ as matrices $N_c\times N_c$ with indices ${{\tilde{n}}_1,{\tilde{n}}_2}$ defined at each $p_\parallel$ site $(n_1,n_2,n_3,n_4)$ (see again Appendix C). Then the non-diagonal terms become diagonal when shifting iteratively *v* by *L* / 2 in the $p_\parallel$ space. Incidentally, we must consider *L* to be even so that at the same time *L* / 2 is well defined and (in the massless case) no spurious pole is hit when Eq. (35) is evaluated in finite volume: this stems from the fact that the staggered action is only invariant under translation of two lattice spacings, therefore twisted boundary conditions would be inconsistent for *L* odd.

## The critical mass of Wilson fermions

The inverse of the Wilson fermion propagator in momentum space can be expressed as

36 $$\begin{aligned} a\Gamma (ap,am,\beta ^{-1}) =\,&aS(ap,am,\beta ^{-1})^{-1} \nonumber \\ =\,&i\sum _\mu \gamma _\mu \overline{(ap_\mu )}+\frac{1}{2}\widehat{(ap)}^2\nonumber \\&+am- a\Sigma (ap,am,\beta ^{-1})\,, \end{aligned}$$

where ${\bar{v}}_\mu = \sin v_\mu$, ${\hat{v}}_\mu = 2\sin (\frac{v_\mu }{2})$ and $\Sigma (ap,am,\beta ^{-1})$ is the self energy. In this section the lattice spacing *a* is written explicitly. Wilson fermions are not equipped with chiral symmetry when the bare mass *m* vanishes: the self energy at zero momentum is affected by a power divergence $a^{-1}$, which has to be cured by an additive renormalisation. In an on-shell renormalisation scheme, the critical value of the bare mass, $m_c$, for which the lattice theory describes massless fermions, is given by the solution of

37 $$\begin{aligned} am_c - a\Sigma (ap=0,am_c,\beta ^{-1}) = 0\,. \end{aligned}$$

As observed in Ref. [36], this prescription matches the one obtained by requiring the chiral Ward identity to hold in the continuum limit. Expanding Eq. (37) defines the critical mass order by order in perturbation theory. The perturbative expansion of the inverse propagator is

38 $$\begin{aligned} a\Gamma (ap,am,\beta ^{-1}) = \sum _{n=0} \Gamma ^{(n)}\left( ap, am \right) \beta ^{-n} \, , \end{aligned}$$

where we have indicated explicitly the dependence of the coefficients on the bare mass *am*. The functions $\Gamma ^{(n)}(ap,am)$ are matrices in Dirac space; since we are interested in the small momentum region and $\Gamma ^{(n)}(0,am)$ is proportional to the identity, we consider $\Gamma ^{(n)}(ap,am)$ as scalar functions: when $ap\ne 0$ a projection onto the identity is understood. Plugging the perturbative expansion of the critical mass

39 $$\begin{aligned} am_c = \sum _{n=1} m_c^{(n)} \beta ^{-n} \end{aligned}$$

into Eq. (38) results in

40 $$\begin{aligned} a\Gamma (ap,am_c,\beta ^{-1})= & {} \sum _{n=0} \gamma ^{(n)}\left( ap\right) \beta ^{-n} \nonumber \\= & {} \sum _{n=0} \left[ m_c^{(n)}+\chi ^{(n)}\left( ap\right) \right] \beta ^{-n} \, , \end{aligned}$$

where the dependence of $\gamma ^{(n)}$ on $m_c^{(n)}$ has been made explicit and $\chi ^{(n)}$ depends only on $m_c^{(0)},\dots ,m_c^{(n-1)}$. Therefore, the renormalisation condition in Eq. (37) becomes order by order

41 $$\begin{aligned} \gamma ^{(n)}(0)=0 \qquad \text {or}\qquad m_c^{(n)}=-\chi ^{(n)}(0)\,. \end{aligned}$$

For illustration, we can compute the recursive solution of Eq. (37) at first- and second-order in the expansion in powers of $\beta ^{-1}$, which yields

42a $$\begin{aligned}&\gamma ^{(1)}(0)= \Gamma ^{(1)}(0,0) + m_c^{(1)} = 0 \, , \end{aligned}$$

42b $$\begin{aligned}&\gamma ^{(2)}(0) = m^{(1)}_c \,\frac{\partial \Gamma ^{(1)}}{\partial (am)}\bigg |_{ap=0,am=0}+ \Gamma ^{(2)}(0,0) + m_c^{(2)}= 0\, . \end{aligned}$$

Both results are familiar from analytical calculations of the critical mass. The first equation encodes the fact that the mass counterterm at first order in perturbation theory is given by the one-loop diagrams computed at zero bare mass. The second equation states that the second-order correction is given by summing two-loop diagrams evaluated at vanishing bare mass, and one-loop diagrams with the insertion of the $O\left( \beta ^{-1}\right)$ counterterm, see e.g. Ref. [37].

It should also be noted that, when working in finite volume, momenta are quantised. Unless periodic boundary conditions are used, $p=0$ is not an allowed value for the momentum of the states in a box. Therefore, condition (37) can only be imposed after extrapolating the value of $\Sigma$ to vanishing momentum. The detailed implementation is discussed below in Sect. 5.1.

Critical masses have been computed analytically up to two loops [37, 38], and in NSPT at three and four loops [39, 40]. High-order perturbation theory with massless Wilson fermions requires the tuning of the critical mass at the same order in $\beta ^{-1}$, and it is possible to determine this renormalisation using NSPT. Let us illustrate the strategy in detail. We begin by collecting configurations for different time steps $\tau$ of the stochastic process; for each configuration the gauge is fixed to the Landau gauge [41, 42]. The propagator at momentum *p* is computed by applying the inverse Dirac operator to a point source in momentum space,

43 $$\begin{aligned} S(p)_{\alpha \beta }= \left\langle \sum _{q\gamma } M\left[ U\right] ^{-1}_{pq,\alpha \gamma }\delta _{qp}\delta _{\gamma \beta }\right\rangle _\text {MC}\,. \end{aligned}$$

For each simulation at a given value of $\tau$, the error bars are computed as detailed in Appendix D. The propagator with periodic boundary conditions is a (diagonal) matrix in colour and momentum space and has a Dirac structure; it is important to stress again that with TBC there is not a colour structure any more and the momentum has a finer quantisation. The average over all the configurations gives the Monte Carlo estimate of *S*(*p*). We can now extrapolate the stochastic time step to zero and invert the propagator to obtain $S(p)^{-1}$. Finally, the inverse propagator is projected onto the identity in Dirac space. All these operations are performed order by order in perturbation theory keeping in mind that, after the measure of the propagator, all perturbative orders $\beta ^{-k/2}$ with an odd *k* are discarded, since the expansion in powers of $\beta ^{-1/2}$ is an artefact of NSPT. The errors can be estimated by bootstrapping the whole procedure.

The legacy of this process is the measure of the functions $\gamma ^{(n)}(ap)$, as it is clear from Eq. (40). The renormalisation condition in Eq. (41) must then be imposed: this can be done iteratively one order after the other. When all the coefficients up to some $m_c^{(n)}$ are included in the simulation, all the $\gamma$ functions up to $\gamma ^{(n)}(ap)$ extrapolate to zero; on the other hand, from $\gamma ^{(n+1)}(0)$ we can read $-m_c^{(n+1)}$. In order to move on and compute the following coefficient of the critical mass, a new set of configurations where $m_c^{(n+1)}$ is taken into account must be generated.

The procedure we described is well defined and even theoretically clean, since it enlightens the status of our $m_c$ as a perturbative additive renormalisation: once it is plugged in at a given order, the renormalised mass turns out to be zero at the prescribed order. On the other side, it is not at all the only possible procedure. The prescription of the authors of Ref. [23] is to expand the solution of the stochastic process both in the coupling and in the mass counterterm. This is in the same spirit of Ref. [43]: the solution of the stochastic process can be expanded in more than one parameter and once a precise power counting is in place, the resulting hierarchy of equations can be exactly truncated at any given order. There are pros and contras for both approaches, i.e. the one we followed and the double expansion. The latter can provide a better handle on estimating errors due to the critical mass value; on the other side, it is expected to be numerical more demanding. All in all, we did not push Wilson fermions to very high orders: moving to the staggered formulation was by far the most natural option for the purpose of this work.

### Zero-momentum extrapolation and valence twist

Since in finite volume it is possible to measure $\Gamma (ap)$ only for discretised non-zero momenta, the data need to be extrapolated to zero momentum using a suitable functional form. The strategy adopted in the literature – see for example Eqs. (13) and (14) in Ref. [40] – is based on expanding the quantities of interest in powers of *ap*. In the infinite-volume limit, such an expansion leads to a hypercubic symmetric Taylor expansion composed of invariants in *ap*, logarithms of *ap* and ratios of invariants; an explicit one-loop computation to order $a^2$ is shown e.g. in Eq. (24) of Ref. [44]. The ratios and the logarithms arise because we are expanding a nonanalytic function of the lattice spacing: infrared divergences appear when expanding the integrands in *ap*. On the other hand, working consistently in finite volume does not cause any infrared divergence: expressions for $\gamma ^{(n)}(ap)$ will be just sums of ratios of trigonometric functions, which we can expand in *ap* obtaining simply a combination of polynomial lattice invariants.^4^

Still, this is not enough for a reliable extrapolation to vanishing momenta. In order to understand better the range of momenta that allow a reliable extrapolation, we computed $\gamma ^{(1)}(ap)$ in twisted lattice perturbation theory (see Appendix E). As a cross-check of our calculation we verified that $\gamma ^{(1)}(0)$ is gauge-invariant (this result must be true at all orders because of the gauge-invariance of the pole mass [45]). It can be seen from the analytic expansion of $\gamma ^{(1)}(ap)$ that even the lowest momentum allowed on our finite-size lattices, $ap_{1,2,3}=0$, $ap_4 = \pi /L$, is far from the convergence region of this series. This happens even for reasonably big lattices, $L\lesssim 32$. In order to increase the range of available momenta, we use $\theta$-boundary conditions [46] for the valence fermions,

44 $$\begin{aligned} \psi (x+L\hat{4})=e^{i\theta }\psi (x)\,, \end{aligned}$$

thereby reaching momenta $p_4 = \theta /L$ which are within the convergence radius of the *ap*-expansion. The hypercubic series becomes just a polynomial in $(ap_4)^2$ by setting all the other components to zero.

**Fig. 1:**
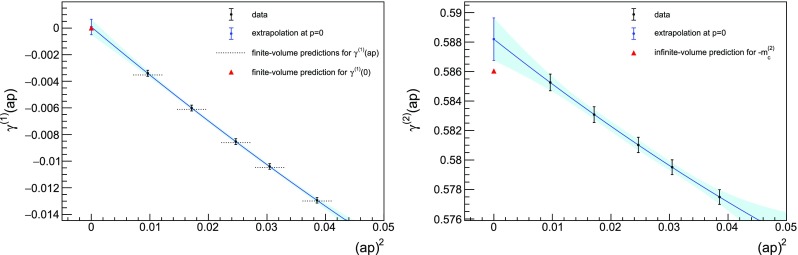
Measure of $\gamma ^{(1)}(ap)$ (left panel) and $\gamma ^{(2)}(ap)$ (right panel) for a $12^4$ lattice with twisted boundary conditions on a plane, $N_c = 2$ and $N_f = 2$ Wilson fermions. The analytic finite-volume critical mass $m_c^{(1)}$ is included in the simulation. A second-order polynomial in $(ap)^2$ is used for fitting. Most analytic finite-volume predictions have been drawn as lines to help the eye in the comparison. The difference with the prediction in the right panel is to be ascribed to the fact that we are able to resolve finite volume effects

**Fig. 2:**
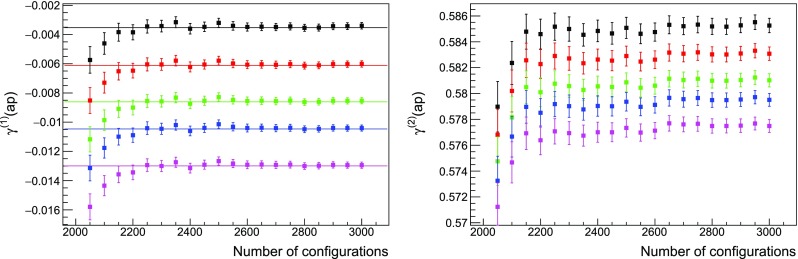
Same as Fig. 1 with data drawn as a function of the number of configurations included in the analysis. Each colour corresponds to a different momentum. Horizontal lines are the analytical predictions

The agreement between data and the analytic finite-volume calculations can be seen in Fig. 1. It is worthwhile to emphasise that measuring such low momenta requires a careful analysis of the thermalisation. At the lowest order we can check directly when the measures agree with the theoretical predictions. At higher orders, it is necessary to wait until the statistical average has clearly stabilised, as shown in Fig. 2. This kind of analysis is computationally intensive: in the case at hand, we performed up to $5 \cdot 10^6$ lattice sweeps, saving one propagator every $10^3$ sweeps. The first $2 \cdot 10^3$ configurations have been discarded in the analysis.

### A first attempt for high-order critical mass for SU(3), $N_f = 2$

We determined the first 7 coefficients of the critical mass for $N_c = 3$ and $N_f = 2$ on a $16^4$ lattice with twisted boundary conditions on a plane. The twist matrices are

45 $$\begin{aligned} \Omega _1= \begin{pmatrix} e^{-i\frac{2\pi }{3}} &{} 0 &{} 0 \\ 0 &{} 1 &{} 0 \\ 0 &{} 0 &{} e^{i\frac{2\pi }{3}} \end{pmatrix} \qquad \Omega _2= \begin{pmatrix} 0 &{} 1 &{} 0 \\ 0 &{} 0 &{} 1 \\ 1 &{} 0 &{} 0 \end{pmatrix}\,, \end{aligned}$$

corresponding to $z_{12}=\exp {\left( i\frac{2\pi }{3}\right) }$. Configurations are collected at three different time steps, $\tau =0.005$, 0.008, 0.01. Because the volume and the number of colours are large compared to the former test in Fig. 1, it is computationally too expensive to replicate the same statistics at all orders: we settled for $5\cdot 10^5$ sweeps at the smallest $\tau$, measuring the propagator every $r=10^3$ sweeps. At larger time steps, we rescale these numbers to keep the product $r\cdot \tau$ constant. The propagator is measured at the smallest available momentum, which has $\theta /L$ in the time component and vanishes elsewhere; we choose three different values for the phase of the valence twist, $\theta =\pi /2$, $2\pi /3$, $4\pi /5$. Extrapolations to zero momentum are performed using a linear fit in $(ap)^2$. The analysis is performed on different subsets of the data^5^ to estimate systematic errors. The total error is the sum in quadrature of half the spread around the central value among the different fits and the largest error from the fits.

**Fig. 3:**
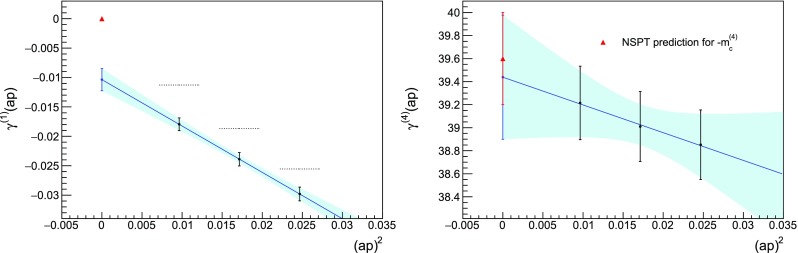
Determination of the coefficient $m_c^{(4)}$. Although $\gamma ^{(1)}(ap)$ does not extrapolate to zero, the extrapolation of $\gamma ^{(4)}(ap)$ is compatible with the value known from Ref. [40]. Notation as in Fig. 1

**Fig. 4:**
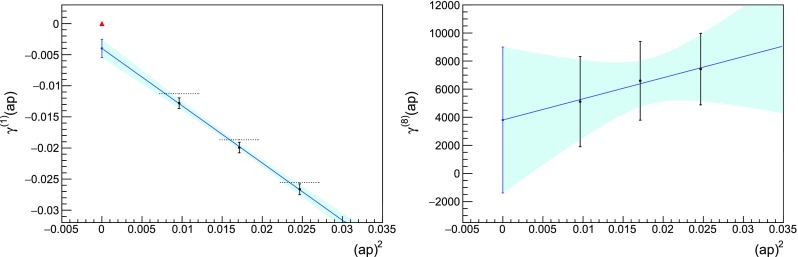
Determination of the coefficient $m_c^{(8)}$. The errors overshadow the value of the critical mass, which is compatible with zero. Notation as in Fig. 1

The procedure described in Sect. 5.1, even though well-defined, is found to be numerically unstable at high orders. The number of propagators required to reach a clear plateau, like the ones shown in Fig. 2, is beyond what it can be reasonably collected with the current NSPT implementations. Therefore, we decided to proceed with a smaller statistics and to add a new systematic uncertainty for the extrapolated coefficients, as explained below. It has to be emphasised that once a coefficient of the critical mass is determined, only the central value is used as input for the following runs: even if we could collect enough statistics and manage to reduce the error, that is not included in the simulations. This makes the impact of the uncertainty of $m_c^{(n)}$ on $m_c^{(n+1)}$ and higher hard to assess; also, performing simulations for several values of each coefficient is not feasible. To be conservative, we adopted the following strategy. Once a critical mass $m_c^{(n)}$ is determined and put in the next-order simulation, the corresponding $\gamma ^{(n)}(ap)$ should extrapolate to zero. If it extrapolates to $\epsilon _n$, we take $|\epsilon _n/m_c^{(n)}|$ as an estimate of the relative systematic error to be added in quadrature to the determination of all the higher-order critical masses.

Despite these instabilities, the lower-order results are close to the known coefficients (keeping in mind that we might resolve finite-volume effects), as it can be seen for example in Fig. 3. We stopped the procedure at $m_c^{(8)}$, when the errors started dominating over the central value of the coefficient, see Fig. 4. Our results are summarised in Table 1.

**Table 1:** Critical masses for $N_c=3$, $N_f=2$ Wilson fermions determined with NSPT on a $16^4$ lattice with twisted boundary condition on a plane, compared with the known values in infinite volume. The $n=1$ coefficient has been determined analytically in twisted lattice perturbation theory; many digits have been used in the actual simulation

*n*	$-m_c^{(n)}$ on $16^4$	$-m_c^{(n)}$ in infinite volume
1	$2.61083\dots$	$2.60571\dots$
2	4.32(3)	4.293(1) [37, 38]
3	$1.21(1)\cdot 10^1$	$1.178(5)\cdot 10^1$ [39, 40]
4	$3.9(2)\cdot 10^1$	$3.96(4)\cdot 10^1$ [40]
5	$1.7(2) \cdot 10^2$	–
6	$5(1) \cdot 10^2$	–
7	$2(1) \cdot 10^3$	–

## Perturbative expansion of the plaquette

Following Ref. [16], we define the average plaquette

46 $$\begin{aligned} P=\frac{1}{6N_cL^4}\sum _\Box {{\mathrm{{\text {Re}}}}}{{\mathrm{\mathrm {Tr}}}}\left( 1-U_\Box \right) \, , \end{aligned}$$

so that the value of *P* ranges between 0, when all link variables are equal to the identity, and 1. The plaquette expectation value has the perturbative expansion

47 $$\begin{aligned} \mathinner {\langle {P}\rangle }_\text {pert}=\sum _{n=0}^\infty p_n\,\beta ^{-(n+1)}\, ; \end{aligned}$$

the coefficients $p_n$ are obtained from the Langevin process.

### Simulation details

We run NSPT simulations of an ${{\mathrm{{\mathrm {SU}}}}}(3)$ gauge theory with $N_f=2$ massless staggered fermions in the fundamental representation, measuring the average plaquette after each Langevin update. Twisted boundary conditions are imposed on a plane, with twist matrices chosen as in Eq. (45). These simulations have been mostly run with the GridNSPT code on KNL and Skylake nodes provided by the Cambridge Service for Data Driven Discovery (CSD3); simulations on the smallest lattice have been run on the Skylake nodes on the Marconi system provided by CINECA in Bologna. The main features of our code are described in Appendix F. We simulate $24^4,28^4,32^4,48^4$ volumes up to order $\beta ^{-40}$ in the expansion of the links. We gradually switch on higher orders when the plaquette at lower orders is thermalised. Because of the instabilities discussed in Sect. 6.2, results are presented only up to the order shown in Table 2. All simulations are run independently at three different time steps, and we have at least $5\cdot 10^3$ measures for the largest order at the smallest time step. The length of the runs at larger time steps is rescaled to have approximately the same Langevin time history for all $\tau$.

**Table 2:** Summary of the ensembles for $N_c=3$ and $N_f=2$ staggered fermions. The order $n_{\text {max}}$ is the highest order at which the plaquette $p_n$ has been measured

*L*	$\tau$	$n_\text {max}$
24	0.005	35
	0.0075	35
	0.01	35
28	0.005	29
	0.008	35
	0.01	35
32	0.005	33
	0.008	35
	0.01	35
48	0.005	35
	0.008	35
	0.01	35

### Numerical instabilities

The study of the NSPT hierarchy of stochastic processes is not trivial. While there are general results for the convergence of the generic correlation function of a finite number of perturbative components of the fields [18, 47], the study of variances is more involved, and many results can only come from direct inspection of the outcome of numerical simulations. In particular, one should keep in mind that in the context of (any formulation of) NSPT, variances are not an intrinsic property of the theory under study; in other words, they are not obtained as field correlators of the underlying theory. Big fluctuations and correspondingly huge variances were observed at (terrifically) high orders in toy models [47]: signals are plagued by several spikes and it is found by inspection that a fluctuation at a given order is reflected and amplified at higher orders. All in all, variances increase with the perturbative order (not surprisingly, given the recursive nature of the equations of motion). Moving to more realistic theories, a robust rule of thumb is that, as expected on general grounds, the larger the number of degrees of freedom, the less severe the problems with fluctuations are. In particular, we have not yet found (nor has anyone else reported) big problems with fluctuations in the computation of high orders in pure Yang–Mills theory.

**Fig. 5:**
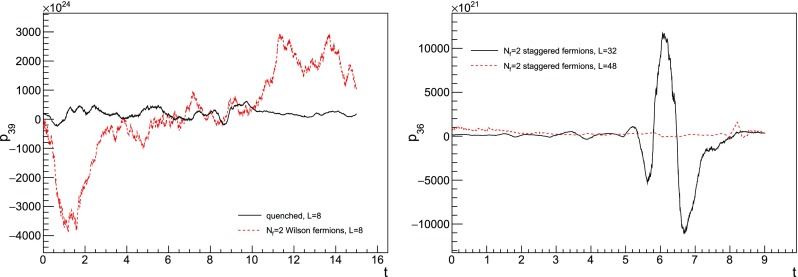
In the left panel, signal samples of the coefficient $p_{39}$ taken from a $8^4$ lattice with TBC in three directions. The simulation with Wilson fermions has been performed for illustrative reasons and the bare mass has been set to zero. In the right panel, signal samples of the coefficient $p_{36}$ with TBC on a plane and staggered fermions. In both panels $\tau =0.005$ and the origin of *t* is set arbitrarily. It is evident that in the quenched case we could extract the plaquette coefficient even from a small volume, while fermions introduce instabilities that can be mitigated by considering bigger lattices. While we have chosen these two particular examples for illustration purposes, the appearance of spikes is a general phenomenon that we observe for orders approximately $\ge 30$ on the volumes under study

We now found that the introduction of fermions indeed causes instabilities at orders as high as the ones we are considering in this work. Once again, this effect can be tamed by working on increasingly large volumes. Once a fluctuation takes place, the restoring force would eventually take the signal back around its average value but in practice this mechanism is not always effective. At high orders the instabilities can be so frequent and large that the signal is actually lost, and the average value of the plaquette becomes negligible compared to its standard deviation, as it is illustrated in Fig. 5. The order at which the signal is lost is pushed to higher values by increasing the volume, but eventually uncontrolled fluctuations will dominate. Moreover, we find that spikes tend to happen more frequently at smaller $\tau$. Roughly speaking, this does not come as a surprise, since at smaller time steps one has to live with a larger number of sweeps, thereby increasing the chances of generating large fluctuations when computing the force fields. In Table 2 the orders available at each volume and time step are shown in detail.

**Fig. 6:**
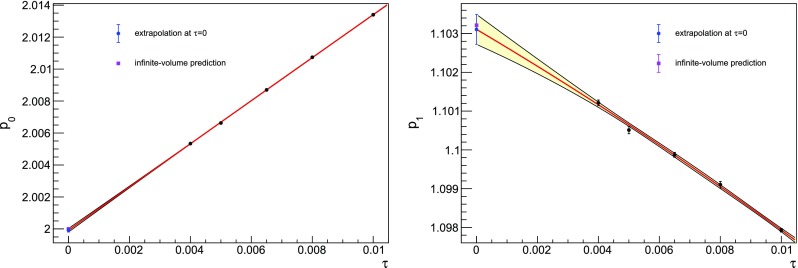
Determination of $p_0$, $p_1$ at $L=48$. Dedicated simulations for these two coefficients have been performed at $\tau =0.004$ and $\tau =0.0065$. We extrapolate to zero time step with a second order polynomial in $\tau$. The extrapolated values are $p_0 = 1.9999(1)$ and $p_1 = 1.1031(4)$ with reduced $\chi ^2$ respectively equal to 1.710 and 1.477

### Determination of the $p_n$

The lowest coefficients have already been computed analytically. In particular, in twisted lattice perturbation theory we have that48is volume independent [48]. The infinite-volume value of $p_1$ can be obtained adding to the pure gauge contribution [49],

49 $$\begin{aligned} p_{1,g}=4N_c^2(N_c^2-1)\left( 0.0051069297-\frac{1}{128N_c^2}\right) \,, \end{aligned}$$

the contribution due to staggered fermions [50],

50 $$\begin{aligned} p_{1,f}= -1.2258(7) \cdot 10^{-3}\,(N_c^2-1)2N_cN_f\,. \end{aligned}$$

For the specific case $N_c=3,N_f=2$, we find $p_1=1.10312(7)$. We also computed the fermion contribution to $p_1$ in twisted lattice perturbation theory.^6^ The finite-volume result is $p_1=1.10317022\dots$ at $L=8$, therefore we expect finite volume effects to be negligible in the lattices we are employing. In particular, we improved the determination of $p_{1,f}$ in Eq. (50) using the finite volume calculations at $L=16$ as the central value, and the variation between $L=16$ and $L=14$ as an estimate of its uncertainty, leading to $p_{1,f}=-0.0587909(3)N_f$ for $N_c=3$, and hence $p_1=1.1032139(6)$ for $N_f=2$. Trying to extract $p_0$ and $p_1$ from our data at $L=48$, we realise that even $\tau ^2$ effects in the extrapolation must be considered because of the very high precision of the measurements. For these two coefficients, a dedicated study at has been performed, which required new simulations at time steps $\tau =0.004$ and $\tau =0.0065$; the agreement with the analytic calculations is found to be excellent, see Fig. 6.

**Fig. 7:**
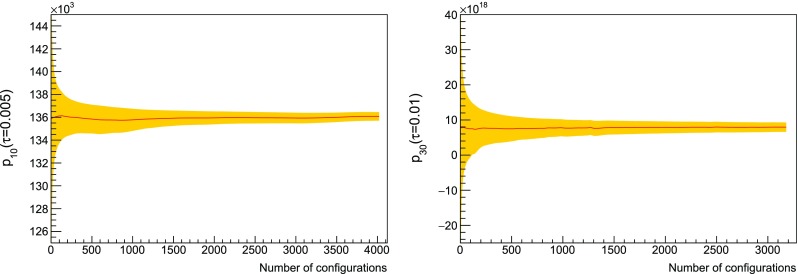
Average of two plaquette coefficients at $L=48$ as a function of the number of configurations. The error band corresponds to the standard deviation of the sample

**Fig. 8:**
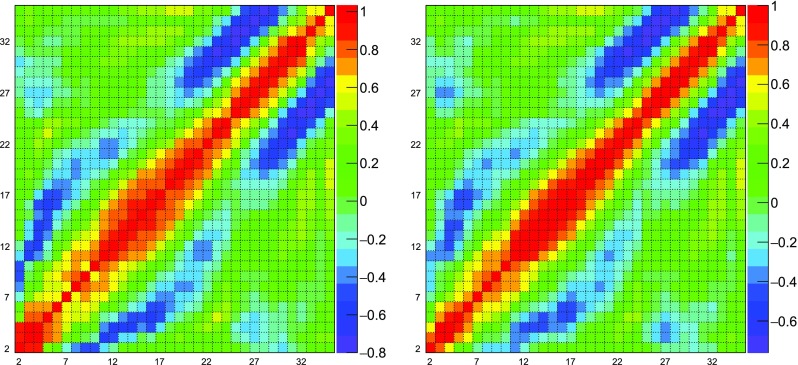
In the left panel, correlation matrix between the coefficients $p_2,\ldots ,p_{35}$ at $L=48$ extracted from the combined fit procedure. The entrances can be bigger than 1 because the matrix is not positive definite. In the right panel, the nearest correlation matrix obtained with Higham’s algorithm ($\delta =10^{-10}$)

Therefore, $p_0$ and $p_1$ are set to their infinite-volume values and excluded from the analysis of the numerical simulations. The remaining orders are obtained from NSPT. The value $p_{n,\tau }$ for the plaquette at order *n* and time step $\tau$ is computed from the average of the fields generated by the stochastic process, after discarding a number of thermalisation steps. The moving averages result to be stable, as can be seen in the two examples of Fig. 7. In order to exploit all the available data, the thermalisation is set differently at different orders. The covariance $\text {Cov}(n,m)_\tau$ between $p_{n,\tau }$ and $p_{m,\tau }$ is computed taking into account autocorrelations and cross-correlations, as explained in detail in Appendix D. Clearly there is no correlation between different $\tau$. In order to estimate the covariance when two orders have different thermalisations, we take into account only the largest set of common values where both are thermalised. This pairwise estimation of the covariance matrix does not guarantee positive definiteness, therefore we rely on Higham’s algorithm, which we describe in Appendix G, to find the nearest positive definite covariance matrix; the procedure introduces some dependence on a tolerance $\delta$. The extrapolation to vanishing time step is performed by minimising

51 $$\begin{aligned} \chi ^2= & {} \sum _{n,m}^{n_{max}}\sum _{\tau } (p_{n,\tau }-a_n\tau -p_n)\, \text {Cov}^{-1}(n,m)_\tau \,\nonumber \\&\qquad \times (p_{m,\tau }-a_m\tau -p_m)\,, \end{aligned}$$

where the coefficients $a_n$ are the slopes of the combined linear fits. The interesting fit results are the values of the extrapolated plaquettes $p_n$ and their covariance matrix $\text {Cov}(n,m)$. In general, because of the available statistics and the intrinsic fluctuations of the observable, the lower-order values are measured more accurately compared to the higher-order ones; the same holds for the estimate of the entries the covariance matrix. Since, in principle, the plaquette at a certain order could be extracted without any knowledge about its higher-order values, we can get the best estimate for a $p_n$ by implementing the fit iteratively, increasing $n_{max}$ from 0 to the maximum available order. At each iteration, we determine the order with the minimum number of measures $N_\text {min}$ and rescale the entries of the covariance matrix so that there is a common normalisation ($N=N_\text {min}$ in Eq. (83)) for all the matrix elements. In this way, all the data are exploited for the determination of the covariance of the process, and the non-positive definiteness of the covariance of the averages arises only from the presence of autocorrelations and cross-correlations. Higham’s algorithm is then applied to $\text {Cov}(n,m)_\tau$ restricted to $n_{max}$ orders. At this stage, minimising the $\chi ^2$ allows us to extract $p_{n_{max}}$ with $\text {Cov}(n_{max},m)$ for $m\le n_{max}$. The tolerance of Higham’s algorithm is tuned so that the covariance matrix is able to represent our data, i.e. so that the reduced chi-squared is close to 1. The combined fit determines also the plaquettes at orders lower than $n_{max}$, which are always checked and found to be in agreement, within errors, with their previous determination at smaller $n_{max}$. An example of a correlation matrix extracted with this procedure is in Fig. 8, where clear structures of correlated and anticorrelated coefficients are visible. The results of the combined extrapolations are summarised in Table 3.

**Table 3:** Plaquette coefficients from the combined fit for $L=24$, 28, 32, 48. The tolerance $\delta$ is given only when the covariance matrix is found not to be positive definite

$L=24$				$L=28$			
*n*	$p_n$	$\chi ^2/\text {dof}$	$\delta$	*n*	$p_n$	$\chi ^2/\text {dof}$	$\delta$
2	2.536(1)	2.178	−	2	2.537(1)	0.032	−
3	7.622(6)	1.079	0.1	3	7.639(7)	1.136	0.625
4	$2.626(3) \cdot 10^{1}$	0.735	0.1	4	$2.636(3) \cdot 10^{1}$	0.648	0.5
5	$9.84(1) \cdot 10^{1}$	0.615	0.1	5	$9.89(2) \cdot 10^{1}$	0.853	0.1
6	$3.906(6) \cdot 10^{2}$	0.828	0.01	6	$3.934(7) \cdot 10^{2}$	0.593	0.1
7	$1.615(3) \cdot 10^{3}$	0.529	0.01	7	$1.630(4) \cdot 10^{3}$	0.480	0.1
8	$6.89(2) \cdot 10^{3}$	0.581	0.01	8	$6.97(2) \cdot 10^{3}$	0.707	0.1
9	$3.021(9) \cdot 10^{4}$	0.421	0.01	9	$3.05(1) \cdot 10^{4}$	0.927	0.1
10	$1.357(5) \cdot 10^{5}$	0.861	0.01	10	$1.366(5) \cdot 10^{5}$	0.753	0.1
11	$6.09(3) \cdot 10^{5}$	0.940	0.01	11	$6.21(3) \cdot 10^{5}$	0.599	0.1
12	$2.80(2) \cdot 10^{6}$	0.753	0.01	12	$2.87(1) \cdot 10^{6}$	0.512	0.1
13	$1.302(9) \cdot 10^{7}$	0.690	0.01	13	$1.338(7) \cdot 10^{7}$	0.443	0.1
14	$6.14(4) \cdot 10^{7}$	0.570	0.01	14	$6.31(4) \cdot 10^{7}$	0.401	0.1
15	$2.94(2) \cdot 10^{8}$	0.652	0.01	15	$3.01(2) \cdot 10^{8}$	0.360	0.1
16	$1.41(1) \cdot 10^{9}$	0.797	0.01	16	$1.44(1) \cdot 10^{9}$	1.012	0.01
17	$6.79(6) \cdot 10^{9}$	0.758	0.01	17	$6.96(7) \cdot 10^{9}$	0.998	0.01
18	$3.31(3) \cdot 10^{10}$	0.730	0.01	18	$3.36(3) \cdot 10^{10}$	0.972	0.01
19	$1.65(2) \cdot 10^{11}$	0.678	0.01	19	$1.63(2) \cdot 10^{11}$	0.953	0.01
20	$8.3(1) \cdot 10^{11}$	0.732	0.01	20	$8.0(1) \cdot 10^{11}$	0.884	0.01
21	$4.15(7) \cdot 10^{12}$	0.755	0.01	21	$3.89(6) \cdot 10^{12}$	0.829	0.01
22	$2.08(5) \cdot 10^{13}$	0.590	0.1	22	$1.91(3) \cdot 10^{13}$	0.821	0.01
23	$10.0(4) \cdot 10^{13}$	0.569	0.1	23	$9.5(2) \cdot 10^{13}$	0.873	0.01
24	$5.0(2) \cdot 10^{14}$	0.543	0.1	24	$4.7(1) \cdot 10^{14}$	0.851	0.01
25	$2.5(1) \cdot 10^{15}$	0.485	0.1	25	$2.34(6) \cdot 10^{15}$	0.764	0.01
26	$1.34(4) \cdot 10^{16}$	1.140	0.01	26	$1.14(3) \cdot 10^{16}$	0.695	0.01
27	$6.6(2) \cdot 10^{16}$	1.054	0.01	27	$5.7(2) \cdot 10^{16}$	0.687	0.01
28	$3.2(2) \cdot 10^{17}$	0.479	0.1	28	$2.8(1) \cdot 10^{17}$	0.671	0.01
29	$1.6(1) \cdot 10^{18}$	1.124	0.01	29	$1.5(1) \cdot 10^{18}$	0.462	0.01
30	$7.6(7) \cdot 10^{18}$	0.836	0.01	30	$7.1(7) \cdot 10^{18}$	0.855	0.001
31	$3.6(6) \cdot 10^{19}$	0.456	0.01	31	$4.2(7) \cdot 10^{19}$	0.663	0.001
32	$1.8(4) \cdot 10^{20}$	0.443	0.01	32	$2.0(4) \cdot 10^{20}$	0.661	0.001
33	$9(3) \cdot 10^{20}$	0.445	0.01	33	$10(3) \cdot 10^{20}$	0.651	0.001
34	$5(2) \cdot 10^{21}$	0.432	0.01	34	$4(2) \cdot 10^{21}$	0.516	0.001
35	$3(1) \cdot 10^{22}$	0.425	0.01	35	$2(1) \cdot 10^{22}$	0.519	0.001

$L=32$				$L=48$			
*n*	$p_n$	$\chi ^2/\text {dof}$	$\delta$	*n*	$p_n$	$\chi ^2/\text {dof}$	$\delta$
2	2.5370(8)	0.249	−	2	2.5354(7)	2.745	−
3	7.627(4)	1.182	−	3	7.615(3)	1.454	0.01
4	$2.633(2) \cdot 10^{1}$	2.412	−	4	$2.623(1) \cdot 10^{1}$	1.428	0.1
5	$9.882(9) \cdot 10^{1}$	1.378	0.5	5	$9.826(6) \cdot 10^{1}$	1.673	0.1
6	$3.926(5) \cdot 10^{2}$	1.015	0.1	6	$3.897(3) \cdot 10^{2}$	1.653	0.1
7	$1.626(2) \cdot 10^{3}$	0.730	0.1	7	$1.613(2) \cdot 10^{3}$	1.338	0.1
8	$6.96(1) \cdot 10^{3}$	0.929	0.01	8	$6.88(1) \cdot 10^{3}$	1.194	0.1
9	$3.050(6) \cdot 10^{4}$	0.772	0.01	9	$3.007(6) \cdot 10^{4}$	1.079	0.1
10	$1.367(4) \cdot 10^{5}$	0.638	0.01	10	$1.341(3) \cdot 10^{5}$	0.998	0.1
11	$6.22(2) \cdot 10^{5}$	0.963	0.01	11	$6.08(1) \cdot 10^{5}$	0.925	0.1
12	$2.86(1) \cdot 10^{6}$	0.645	0.1	12	$2.793(6) \cdot 10^{6}$	1.108	0.01
13	$1.337(6) \cdot 10^{7}$	0.771	0.1	13	$1.297(3) \cdot 10^{7}$	0.978	0.01
14	$6.29(3) \cdot 10^{7}$	0.861	0.1	14	$6.08(2) \cdot 10^{7}$	0.883	0.01
15	$3.00(2) \cdot 10^{8}$	0.952	0.1	15	$2.87(1) \cdot 10^{8}$	1.067	0.01
16	$1.438(9) \cdot 10^{9}$	1.012	0.1	16	$1.370(5) \cdot 10^{9}$	1.013	0.01
17	$6.94(5) \cdot 10^{9}$	0.996	0.1	17	$6.57(3) \cdot 10^{9}$	0.951	0.01
18	$3.34(3) \cdot 10^{10}$	1.000	0.1	18	$3.16(1) \cdot 10^{10}$	0.930	0.01
19	$1.63(2) \cdot 10^{11}$	0.965	0.1	19	$1.530(6) \cdot 10^{11}$	0.938	0.01
20	$7.90(8) \cdot 10^{11}$	1.053	0.01	20	$7.45(3) \cdot 10^{11}$	0.890	0.01
21	$3.86(4) \cdot 10^{12}$	0.995	0.01	21	$3.65(1) \cdot 10^{12}$	0.824	0.01
22	$1.90(2) \cdot 10^{13}$	0.957	0.01	22	$1.796(9) \cdot 10^{13}$	0.748	0.01
23	$9.4(1) \cdot 10^{13}$	0.949	0.01	23	$8.88(5) \cdot 10^{13}$	0.691	0.01
24	$4.74(9) \cdot 10^{14}$	0.979	0.01	24	$4.41(3) \cdot 10^{14}$	0.636	0.01
25	$2.39(5) \cdot 10^{15}$	0.967	0.01	25	$2.19(2) \cdot 10^{15}$	0.575	0.01
26	$1.22(3) \cdot 10^{16}$	0.921	0.01	26	$1.09(1) \cdot 10^{16}$	0.548	0.01
27	$6.3(2) \cdot 10^{16}$	0.871	0.01	27	$5.46(9) \cdot 10^{16}$	0.538	0.01
28	$3.2(1) \cdot 10^{17}$	0.849	0.01	28	$2.74(6) \cdot 10^{17}$	0.523	0.01
29	$1.63(9) \cdot 10^{18}$	0.812	0.01	29	$1.38(4) \cdot 10^{18}$	0.511	0.01
30	$8.6(7) \cdot 10^{18}$	0.779	0.01	30	$7.0(3) \cdot 10^{18}$	0.492	0.01
31	$4.5(9) \cdot 10^{19}$	0.743	0.01	31	$3.5(2) \cdot 10^{19}$	0.494	0.01
32	$1.9(3) \cdot 10^{20}$	0.723	0.01	32	$1.7(1) \cdot 10^{20}$	0.503	0.01
33	$9(2) \cdot 10^{20}$	0.723	0.01	33	$8.3(7) \cdot 10^{20}$	1.062	0.001
34	$5(1) \cdot 10^{21}$	0.702	0.01	34	$5.2(6) \cdot 10^{21}$	1.090	0.001
35	$1(1) \cdot 10^{22}$	0.663	0.01	35	$2.3(6) \cdot 10^{22}$	0.486	0.01

## Gluon condensate

In this section we restore the lattice spacing *a* and follow the notation of Refs. [16, 17]: the gluon condensate is defined as the vacuum expectation value of the operator

52 $$\begin{aligned} O_G=-\frac{2}{\beta _0} \frac{\beta (\alpha )}{\alpha }\sum _{a,\mu ,\nu }G_{\mu \nu }^a G_{\mu \nu }^a\,, \end{aligned}$$

where the coupling $\alpha$ is related to the Wilson action coupling by $\alpha =\frac{N_c}{2\pi \beta }$ and the beta function is

53 $$\begin{aligned} \beta (\alpha )=\frac{d\alpha }{d\ln \mu }=-2\alpha \left[ \beta _0\frac{\alpha }{4\pi }+\beta _1\left( \frac{\alpha }{4\pi }\right) ^2+\dots \right] \,, \end{aligned}$$

with the scheme-independent coefficients

54a $$\begin{aligned} \beta _0&=\frac{11}{3} N_c-\frac{2}{3}N_f \end{aligned}$$

54b $$\begin{aligned} \beta _1&=\frac{34}{3}N_c^2-N_f\left( \frac{13}{3}N_c-\frac{1}{N_c}\right) \,. \end{aligned}$$

It is useful to remember that, in the massless limit, $O_G$ is renormalisation group invariant and depends on the scheme only through the renormalisation condition used to define the composite operator.

It is easy to relate the gluon condensate and the plaquette in the naive continuum limit:

55a $$\begin{aligned}&a^{-4}P \xrightarrow {a\rightarrow 0} \frac{\pi ^2}{12N_c}O_G=\frac{\pi ^2}{12N_c}\left( \frac{\alpha }{\pi }G^2 \right) \,, \end{aligned}$$

55b $$\begin{aligned}&O_G= \frac{\alpha }{\pi }G^2 \left[ 1+O(\alpha ) \right] \,. \end{aligned}$$

In the interacting theory mixing with operators of lower or equal dimension occurs. For the case of the plaquette, the mixing with the identity needs to be considered, yielding

56 $$\begin{aligned} a^{-4}P = a^{-4}Z(\beta ){\mathbb {1}}+\frac{\pi ^2}{12N_c}C_G(\beta )O_G+O(a^2\Lambda _\text {QCD}^6)\, , \end{aligned}$$

which shows explicitly the subtraction of the quartic power divergence.^7^

As a consequence

57 $$\begin{aligned} \mathinner {\langle {P}\rangle }_\text {MC} = Z(\beta ) + \frac{\pi ^2}{12N_c} C_G(\beta ) a^4 \mathinner {\langle {O_G}\rangle } + O(a^6\Lambda _\text {QCD}^6)\,,\nonumber \\ \end{aligned}$$

where $\mathinner {\langle {P}\rangle }_\text {MC}$ is the plaquette expectation value obtained from a nonperturbative Monte Carlo simulation. As such, $\mathinner {\langle {P}\rangle }_\text {MC}$ is expected to depend on the cut-off scale *a*, and $\Lambda _\text {QCD}$. In the limit $a^{-1}\gg \Lambda _\text {QCD}$, Eq. (57) can be seen as an Operator Product Expansion (OPE) [1, 2, 53], which factorises the dependence on the small scale *a*. In this framework,^8^ condensates like $\mathinner {\langle {O_G}\rangle }$ are process-independent parameters that encode the nonperturbative dynamics, while the Wilson coefficients are defined in perturbation theory,

58 $$\begin{aligned} Z(\beta )=\sum _{n=0}p_n\beta ^{-(n+1)}\,, \quad C_G(\beta )=1+\sum _{n=0}c_n\beta ^{-(n+1)}\,.\nonumber \\ \end{aligned}$$

Note that both *Z* and $C_G$ depend only on the bare coupling $\beta ^{-1}$, and do not depend on the renormalisation scale $\mu$, as expected for both coefficients [55, 56]. Nonperturbative contributions to *Z*, or $C_G$, originating for example from instantons, would correspond to subleading terms in $\Lambda _\text {QCD}$. This procedure defines a renormalisation scheme to subtract power divergences: condensates are chosen to vanish in pertubation theory or, in other words, they are normal ordered in the perturbative vacuum. This definition matches the one that is natural in dimensional regularisation, where power divergences do not arise. Nevertheless, it is well known that such a definition of the condensates might lead to ambiguities, since the separation of scales in the OPE does not necessarily correspond to a separation between perturbative and nonperturbative physics (see the interesting discussions in Refs. [3, 57]). For example, the fermion condensate in a massless theory is well-defined since, being the order parameter of chiral symmetry breaking, it must vanish in perturbation theory. The same cannot be said for the gluon condensate [58], and indeed the ambiguity in its definition is reflected in the divergence of the perturbative expansion of the plaquette. For this picture to be consistent, it must be possible to absorb in the definition of the condensate the ambiguity in resumming the perturbative series.

In the following, we are going to study the asymptotic behaviour of the coefficients $p_n$ determined in the previous section and discuss the implications for the definition of the gluon condensate in massless QCD.

### Growth of the coefficients

**Fig. 9:**
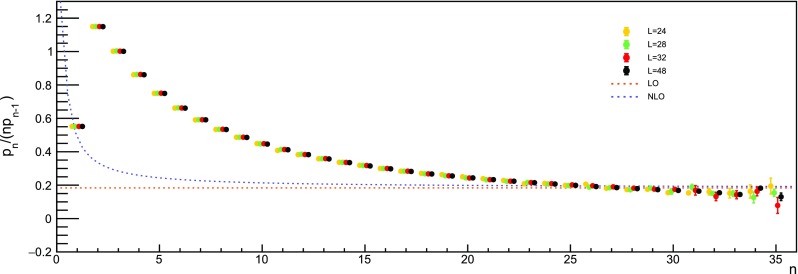
Ratio $p_n/(np_{n-1})$ extracted from our data at $L=24$, 28, 32, 48. In order to be visible, points referring to different volumes are placed side by side. The leading order (LO) prediction refers to the $n\rightarrow \infty$ limit, while the next-to-leading order (NLO) one includes the first 1 / *n* correction

**Fig. 10:**
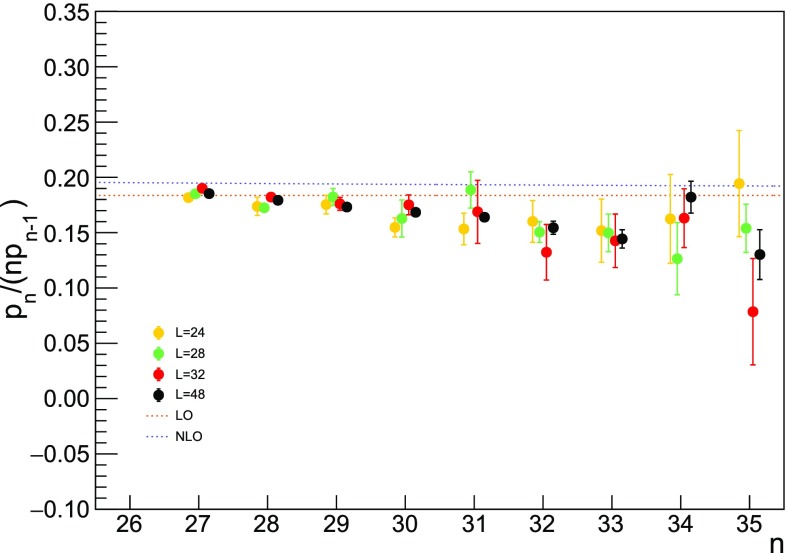
Same as Fig. 9, but the region at large *n* is enlarged

From the analysis in Refs. [11, 16], it is possible to predict the asymptotic behaviour of the ratio

59 $$\begin{aligned} \frac{p_n}{np_{n-1}} = \frac{3\beta _0}{16\pi ^2} \left[ 1+\frac{2\beta _1}{\beta _0^2}\frac{1}{n} + O\left( \frac{1}{n^2}\right) \right] \,, \end{aligned}$$

where the use of the Wilson action with $N_c=3$ is assumed. This relation can be derived under the hypothesis that the plaquette series has a fixed-sign factorial divergence and the corresponding singularity in the Borel plane is the source of an ambiguity that can be absorbed by redefining the condensate. It is not possible to go further in the 1 / *n* expansion since the $\beta _2$ coefficient is scheme-dependent and it is not known for staggered fermions. In Figs. 9 and  10, the comparison between Eq. (59) and our data at different volumes is shown.

How finite-volume effects influence the values of the coefficients $p_n$ has already been studied in the literature [16, 59]. From a standard renormalon-based analysis, the value of the loop momenta that contribute the most to $p_n$ decreases exponentially with *n*. Since the finite size of the lattice provides a natural infrared cutoff, we expect finite-volume effects to be larger at larger perturbative orders. The dependence of $p_n$ on the lattice size *N* can be modelled with a finite-volume OPE, exploiting the separation of scales $a^{-1}\gg (Na)^{-1}$: the leading correction is [16]

60 $$\begin{aligned} \sum _{n=0}p_n(N)\beta ^{-(n+1)}= & {} \sum _{n=0}p_n\beta ^{-(n+1)}\nonumber \\&-\frac{1}{N^4}\,C_G(\beta ) \sum _{n=0}f_n\alpha ((Na)^{-1})^{n+1}\nonumber \\&+O\left( \frac{1}{N^6}\right) \,, \end{aligned}$$

**Fig. 11:**
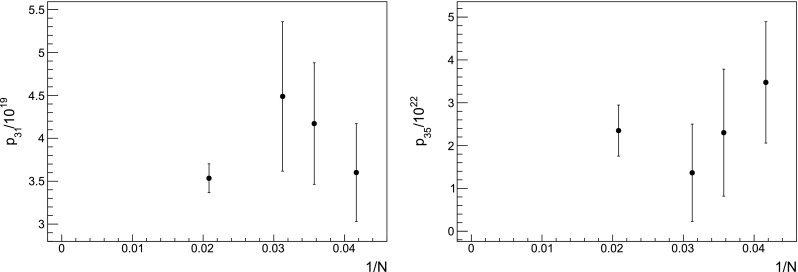
Coefficients $p_{31}$ and $p_{35}$ drawn as a function of the volume. No significant finite-volume effects are observed at our level of precision

where $\alpha ((Na)^{-1})$ must be expressed in terms of the coupling $\beta$ at the scale $a^{-1}$ using Eq. (53). We do not attempt to take into account $1/N^4$ effects, as our data do not allow to perform a reliable combined fit. Apparently no significant finite-volume effects are visible where they would be expected the most, i.e. at larger *n*. This is shown in the two examples of Fig. 11. A similar behaviour has been observed in Ref. [16], where the data points computed on comparable volumes show little dependence on the lattice size. In that study, a detailed analysis with a large number of volumes was needed in order to be able to fit the finite-volume corrections. The overall effect is found to be an increase of the ratio $p_n/(n p_{n-1})$, see e.g. Fig. 6 in Ref. [16]. In our case, data in finite volume do cross the theoretical expectation; still, considering the spread between points at different volumes in Fig. 10 as a source of systematic error, we could consider our measurements to be compatible with the asymptotic behaviour of Eq. (59). We also ascertain the existence of an inversion point when resumming the perturbative series, as explained in Sect. 7.3. Despite this encouraging behaviour, any definite conclusion about the existence of the expected renormalon can only be drawn after performing an appropriate infinite-volume study. We emphasise that in this work the discrepancies in the determination of the $p_ n$ from different volumes must be interpreted as part of our systematic uncertainty, being this an exploratory study. A precise assessment of the finite-volume effects will be sought for a precise determination of the gluon condensate; we are currently planning a set of dedicated simulations in the near future to settle this issue.

**Fig. 12:**
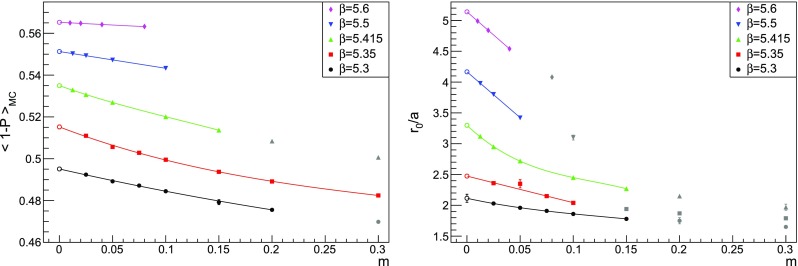
Chiral extrapolation of the nonperturbative plaquette (left panel) and the ratio $r_0/a$ (right panel) at five different values of $\beta$. The grey points are available from Ref. [50] but are excluded because of our fit procedure. In most cases the error bar is smaller than the symbol. The orders of the polynomials used in the fits are in Table 4

### Monte Carlo plaquette

Nonperturbative values for the ${{\mathrm{{\mathrm {SU}}}}}(3)$ plaquette with $N_f=2$ (rooted) staggered fermions can be found in Ref. [50], where data are collected from Refs. [60, 61]. For each value of the bare coupling, the physical scale is provided via the Sommer parameter $r_0$ [62]. The data are given for several values of the fermion bare mass, and need to be extrapolated to the chiral limit for our purposes. A reasonable assumption (for example adopted and verified also in Ref. [63] for the ratio $r_0/a$) is that the plaquette and the ratio $r_0/a$ have a polynomial behaviour at small masses. We performed fits with linear to cubic polynomials and varied the fit ranges to exclude points at larger values of the masses, but in many cases the fits did not return a satisfactory description of the data with sensible values of $\chi ^2/\text {dof}$. Because we are using results from past simulations, it is difficult to track accurately the systematic errors in the data. For this reason, we decided to choose the fit with smaller $\chi ^2/\text {dof}$ among those we tried and if $\chi ^2/\text {dof}>1$ the errors in the data were rescaled by a common factor in order to have a reduced chi-squared equal to 1. The fits resulting from this approach are shown in Fig. 12; the extrapolated values for plaquettes and $r_0/a$ are in Table 4. Another approach consists in considering the average between the largest and smallest extrapolated values among all the different fits we tried (without rescaled errors and with reduced chi-squared smaller than some reasonable threshold) and assigning an error equal to the sum in quadrature between the largest error from the fits and half the difference between the largest and smallest extrapolated values. In this way we obtain results compatible (both for central values and errors) with those in Table 4, confirming that the chiral extrapolation is sound and the error bars conservative enough. Note that in this paper we do not aim at a precise determination of the condensate, and therefore we can be satisfied with an inflated error on the Monte Carlo data points.

**Table 4:** Results of the chiral extrapolation for the plaquette and the scale. The order of the polynomials used in the fits is indicated

$\beta$	$\langle 1-P\rangle _\text {MC}$	Pol. ord.	$r_0/a$	Pol. ord.
5.3	$0.4951\,(4)$	2	$2.11\,(7)$	3
5.35	$0.5152\,(9)$	3	$2.47\,(3)$	1
5.415	$0.5350\,(3)$	3	$3.30\,(3)$	3
5.5	$0.55128\,(3)$	1	$4.17\,(2)$	1
5.6	$0.56526\,(5)$	1	$5.14\,(1)$	1

**Table 5:** Summation up to the minimal term of the perturbative series of the plaquette

$\beta$	*L*	$S_P(\beta )$	${\bar{n}}$	$p_{\bar{n}} \beta ^{-(\bar{n}+1)}$
5.3	24	$0.47515\,(9)$	25	$3.70 \cdot 10 ^{-4}$
	28	$0.4767\,(1)$	30	$2.52 \cdot 10 ^{-4}$
	32	$0.4775\,(4)$	35	$5.23 \cdot 10 ^{-5}$
	48	$0.47665\,(7)$	33	$1.97 \cdot 10 ^{-4}$
5.35	24	$0.46718\,(8)$	25	$2.90 \cdot 10 ^{-4}$
	28	$0.46843\,(9)$	30	$1.88 \cdot 10 ^{-4}$
	32	$0.4690\,(3)$	35	$3.73 \cdot 10 ^{-5}$
	48	$0.46826\,(5)$	33	$1.43 \cdot 10 ^{-4}$
5.415	24	$0.4587\,(1)$	33	$1.06 \cdot 10 ^{-4}$
	28	$0.45844\,(7)$	30	$1.29 \cdot 10 ^{-4}$
	32	$0.4588\,(2)$	35	$2.42 \cdot 10 ^{-5}$
	48	$0.45822\,(4)$	33	$9.51 \cdot 10 ^{-5}$
5.5	24	$0.44663\,(9)$	33	$6.22 \cdot 10 ^{-5}$
	28	$0.44651\,(6)$	30	$7.98 \cdot 10 ^{-5}$
	32	$0.4466\,(1)$	35	$1.38 \cdot 10 ^{-5}$
	48	$0.44627\,(4)$	33	$5.60 \cdot 10 ^{-5}$
5.6	24	$0.43384\,(6)$	34	$3.32 \cdot 10 ^{-5}$
	28	$0.43380\,(5)$	30	$4.57 \cdot 10 ^{-5}$
	32	$0.43383\,(6)$	35	$7.21 \cdot 10 ^{-6}$
	48	$0.43357\,(3)$	33	$3.03 \cdot 10 ^{-5}$

**Fig. 13:**
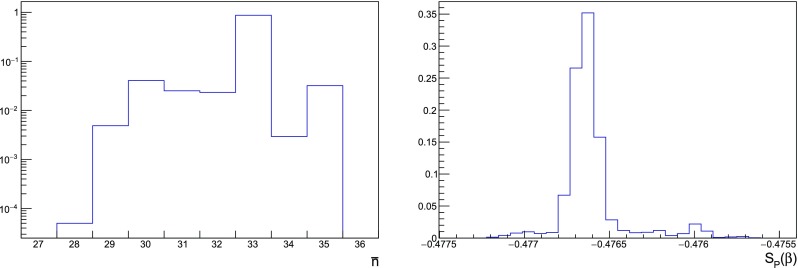
Normalised distributions, over $10^5$ bootstrap samples, of $\bar{n}$ (left panel) and $S_P(\beta )$ (right panel) for $L=48$, $\beta =5.3$

**Fig. 14:**
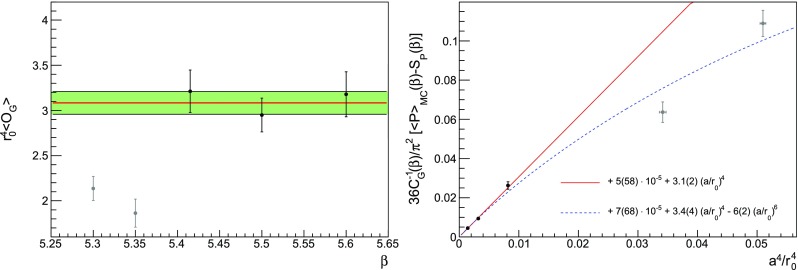
In the left panel, determination of the gluon condensate from Eq. (62). The line corresponds to the weighted average of the three largest values of $\beta$. In the right panel, scaling of the condensate with $a^4$ (solid red line, grey points are excluded), with possibly a $a^6$ correction (dashed blue line, grey points are included). Both panels refer to $L=48$

### Determination of the minimal term

The perturbative contribution to the plaquette can be defined by the sum of the series up to the minimal term. The determination of the minimal term, and the summation of the series are performed separately for each volume. We choose the prescription adopted in Ref. [17], i.e. we define the minimal term to be the value $\bar{n}$ that minimises the product $p_n \beta ^{-(n+1)}$ and resum the series,

61 $$\begin{aligned} S(\beta )_P=\sum _{n=0}^{{\bar{n}}}p_n\beta ^{-(n+1)}\,. \end{aligned}$$

Our results for all combinations of *L* and $\beta$ are summarised in Table 5. The order ${\bar{n}}$ at which the series starts to diverge depends only on the central value of the coefficients $p_n$ and not on their errors: in order to check that the inversion point determined by our procedure is stable, we bootstrapped the procedure by generating an ensemble of sets of coefficients $\left\{ p_n\right\}$. For each set, the coefficients $p_n$ are drawn from a Gaussian probability, whose mean and covariance are taken from the fit procedure described in Sect. 6. We then determine ${\bar{n}}$ for each of these sets. The inversion point turns out to be stable, as shown in Fig. 13 for a the case $L=48$, and $\beta =5.3$. This particular case is shown for illustration purposes, and the same features are seen in all other combinations of *L* and $\beta$.

The gluon condensate is then determined from

62 $$\begin{aligned} \mathinner {\langle {O_G}\rangle }=\frac{36}{\pi ^2} \, C^{-1}_G(\beta )\,a^{-4}\,[\mathinner {\langle {P}\rangle }_\text {MC}(\beta )-S_P(\beta )] \end{aligned}$$

with

63 $$\begin{aligned} C^{-1}_G(\beta )=1+\frac{3}{8\pi ^2}\frac{\beta _1}{\beta _0} \frac{1}{\beta }+O\left( \frac{1}{\beta ^2}\right) \,. \end{aligned}$$

The coefficient $\beta _2$ is not universal, and is actually unknown for the discretisation used in this work. Not knowing $\beta _2$ prevents us from going further in the expansion of $C_G$; since the correction due to the Wilson coefficient falls between $5\%$ and $6\%$ for the values of $\beta$ considered, a $6\%$ systematic uncertainty is added in quadrature after the subtraction.

The result of the subtraction is shown in the left panel of Fig. 14, for the largest volume. Since only a few values of $\beta$ is available, it is hard to assess unambiguously the presence of a plateau. We decided to discard from the analysis the two values of the coupling corresponding to the coarser lattices, and define our best estimate of the condensate as the weighted average of the values obtained at the remaining $\beta$s. Our final results are summarised in the first column of Table 6.

In order to put the choice of fit range on more solid ground, we studied the scaling of $a^4\mathinner {\langle {O_G}\rangle }$ as a function of $a^4$, as shown in Fig. 14. The slope of a linear fit of the three finest lattice spacings should give a determination of the condensate compatible with the value extracted from the weighted average. The spread between these two determinations and among the different volumes gives an idea of the magnitude of the systematic uncertainties involved. We also tried to include in the analysis all the available values of $\beta$ and add a $a^6$ correction, in the attempt to model the deviations at large values of the coupling; this procedure gives again consistent results (despite a larger $\chi ^2$).

Truncating the sum up to the minimal term is one of the possible prescriptions to define the sum of a divergent series. The intrinsic ambiguity associated to $S_P(\beta )$ can be defined as the imaginary part of the Borel integral, which at leading order in 1 / *n* is $\sqrt{\pi {\bar{n}}/2}\,p_{{\bar{n}}}\,\beta ^{-{\bar{n}} -1}$ [5]. In Table 7, the ambiguity associated to the gluon condensate

64 $$\begin{aligned} \delta \mathinner {\langle {O_G}\rangle }=\frac{36}{\pi ^2} \, C^{-1}_G(\beta )\,a^{-4}\, \sqrt{\frac{\pi {\bar{n}}}{2}} \, p_{{\bar{n}}}\beta ^{-{\bar{n}} -1} \end{aligned}$$

is summarised.^9^

**Table 6:** Determination of the gluon condensate at different volumes. The determination labelled with 1 is obtained from the weighted average of the values at the three largest values of $\beta$. The determinations labelled with 2 and 3 are obtained by studying the scaling of $a^4\mathinner {\langle {O_G}\rangle }$ with $a^4$, as in the right panel of Fig. 14; they correspond respectively to the fit without and with $a^6$ correction (see text for the details)

*L*	$r_0^4\mathinner {\langle {O_G}\rangle }_1$	$r_0^4\mathinner {\langle {O_G}\rangle }_2$	$r_0^4\mathinner {\langle {O_G}\rangle }_3$
24	$2.6\,(1)$	$2.9\,(2)$	$3.1\,(4)$
28	$2.8\,(1)$	$3.1\,(2)$	$3.4\,(4)$
32	$2.4\,(1)$	$2.9\,(2)$	$3.2\,(4)$
48	$3.1\,(1)$	$3.1\,(2)$	$3.4\,(4)$

**Table 7:** Ambiguity of the gluon condensate determined from Eq. (64) at the three largest values of $\beta$

*L*	$r_0^4\delta \mathinner {\langle {O_G}\rangle }$		
	$\beta =5.415$	$\beta =5.5$	$\beta =5.6$
24	$0.4\,(2)$	$0.5\,(4)$	$0.7\,(5)$
28	$0.4\,(3)$	$0.7\,(4)$	$0.9\,(5)$
32	$0.3\,(2)$	$0.5\,(3)$	$0.3\,(3)$
48	$0.3\,(2)$	$0.5\,(3)$	$0.6\,(4)$

## Conclusions

We used NSPT to perform for the first time large-order computations in lattice gauge theories coupled to massless fermions. We adopted twisted boundary conditions for the gauge fields to remove the zero-momentum mode. Since our fermions are in the fundamental representation, we consistently provided them with a smell degree of freedom. Both Wilson and (for the first time in NSPT) staggered fermions have been implemented. While for the former we performed an exploratory study of the critical mass up to order $O(\beta ^{-7})$, the latter are ultimately the best choice to reach very high orders, due to their residual chiral symmetry that bypasses the need of an additive mass renormalisation.

Numerical instabilities were noticed in the study of simple models in NSPT since the early days of the method, but gauge theories have always been reported to stay on a safe side in this respect, even at orders as high as the ones we investigated in this work. With fermions in place, we now found that numerical instabilities arise for lattice gauge theories at high orders. While we plan to investigate the causes and develop a solution to this, the problem did not prevent us to reach order $O(\beta ^{-35})$ in the expansion of the basic plaquette for $N_c=3$ and $N_f=2$.

The plaquette has been for a long time the stage for the determination of the gluon condensate, to which is connected in the continuum limit. The perturbative expansion of the plaquette, which corresponds to the power divergent contribution associated to the identity operator in the relevant OPE, must be subtracted from nonperturbative Monte Carlo lattice computations. This long-standing and tough problem was eventually solved a few years ago in pure gauge [16, 17], thanks to NSPT. Equipped with our high-orders expansions, we tackled once again the problem in the lattice regularisation of full QCD. We computed the perturbative expansion of the plaquette, and subtracted it from Monte Carlo measurements. In this context, NSPT is crucial: it is actually the only tool enabling this procedure, which asks for having the asymptotic behaviour of such series under control. This happens since the perturbative expansion of the plaquette is expected to be plagued by renormalon ambiguities. Under the assumption of considering finite-volume effects as a source of systematic errors, the observed growth of the coefficients in the expansion could be compatible with the leading IR renormalon; nevertheless, the large uncertainties and the lack of a study of finite-volume effects prevent us from drawing any definite conclusion. The IR renormalon forces to absorb the ambiguities attached to the perturbative series into the definition of the condensate itself. All in all, this implies that we needed a prescription to perform the computation. The one we chose amounts to summing the perturbative series up to its minimal term (which means computing the series up to orders that only NSPT can aim at).

We regard this project as a first exploratory study. We could confirm both that the IR renormalon can be directly inspected, and that the series can be computed up to orders where the inversion point beyond which the expansion starts to diverge (at values of the coupling which are the typical ones in lattice simulations) is clearly visible. We performed our simulations at different lattice extents, in order to have a first estimate of finite-size effects (again, in both the study of renormalon behaviour and in the truncation of the series). This is the point which has to be better investigated in a following study. At the moment, finite-size effects are still to be considered as a systematic source of errors in our procedure.

On top of the follow-ups we have already discussed, we plan to extend our study to different number of colours, number of flavours and fermionic representations. It would be of the utmost importance to assess the high-order behaviour of perturbative coefficients in gauge theories different from QCD, to probe regions in the space of theories in which a (quasi-)conformal window can be present. This could be a powerful, alternative method to look for candidate theories for physics beyond the Standard Model.

## Figures and Tables

**Fig. 15:**
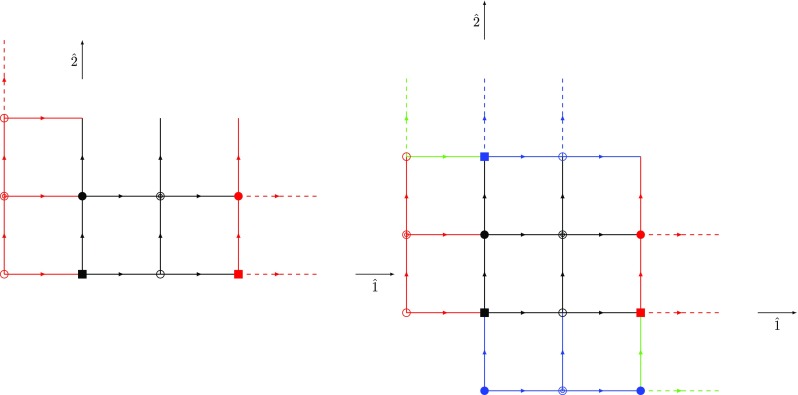
Sketch of the PRlgt auxiliary links in a plane of a $2\times 2$ lattice. Physical links are in black and sites identified by the same symbol represent the same physical site. Dashed lines highlight links that are allocated but do not participate in the update. In the left panel direction ${\hat{1}}$ is twisted, ${\hat{2}}$ is not: in red there are the auxiliary sites (two forward, three backward) and the red links beginning there correspond to physical links twisted according to the matrix $\Omega _1$. In the right panel both ${\hat{1}}$ and ${\hat{2}}$ are twisted directions: in blue there are auxiliary sites whose links are twisted according to the matrix $\Omega _2$. In the latter case there are sites which pass the boundary in two twisted directions: the green links undergo both the $\Omega _1$ and the $\Omega _2$ twist (the two operations commute by definition)

## Group theory conventions

The conventions used for group theoretical manipulations are summarised here. We consider the gauge group ${{\mathrm{{\mathrm {SU}}}}}(N_c)$.

The generators of the group are denoted by $T^a$; the indices $a,b,c=1, \ldots , N_c^2-1$ are assumed to be indices in the adjoint representation. The generators are defined to be Hermitian, and satisfy the commutation relations

65 $$\begin{aligned} \left[ T^a,T^b\right] = \sum _c i f^{abc} T^c\, , \end{aligned}$$

where $f^{abc}$ are the group structure constants. The normalisation of the generators is chosen to be such that

66 $$\begin{aligned} \mathrm {Tr}\left( T^a T^b\right) = \frac{1}{2} \delta ^{ab}\, . \end{aligned}$$

The left derivative on the group is defined as $\nabla _{x\mu }=\sum _a T^a \nabla ^a_{U_{\mu }(x)}$, where the Lie derivative is given by

67 $$\begin{aligned} \nabla ^a_V f(V) = \lim _{\alpha \rightarrow 0} \frac{1}{\alpha } \left[ f\left( e^{i \alpha T^a} V\right) - f(V) \right] \, . \end{aligned}$$

We define an operator, ${{\mathrm{\Pi _\mathfrak {g}}}}$, that projects on the algebra $\mathfrak {g}$ of the group:

68 $$\begin{aligned} {{\mathrm{\Pi _\mathfrak {g}}}}(X) = \frac{1}{2} \left( X - X^\dagger - \frac{1}{N_c} {{\mathrm{\mathrm {Tr}}}}\left( X-X^\dagger \right) \right) \,. \end{aligned}$$

The indices $i,j=1,\ldots ,N_c$ will be used as indices in the fundamental representation, $r,s=1,\ldots ,N_c$ as indices in the antifundamental representation.

## Optimisation of the fermion drift

A useful optimisation consists in improving on Eqs. (29) and (33) so that it becomes numerically cheaper to evaluate the fermion drift. Considering for example Wilson fermions, we notice that it is possible to simplify the trace

69 $$\begin{aligned} {{\mathrm{\mathrm {Tr}}}}(\nabla ^a_{x\mu } M) M^{-1}=\,&i{\tilde{{{\mathrm{\mathrm {Tr}}}}}} \left[ \left( T^aD(\mu )M^{-1}\right) _{x,x}\right. \nonumber \\&\left. -\left( \gamma _5D(\mu )^\dagger \gamma _5T^aM^{-1}\right) _{x,x}\right] \nonumber \\ =\,&i\sum _{y,\beta ,i,r} \left( \delta _{x,y}[T^aD(\mu )M^{-1}]_{y\beta i r, y\beta i r}\right. \nonumber \\&\quad \left. -\text {h.c.}\right) \,, \end{aligned}$$

where ${\tilde{{{\mathrm{\mathrm {Tr}}}}}}$ is tracing all indices but the position one, and we used the fact that the inverse Wilson operator is $\gamma _5$-Hermitian. For staggered fermions the simplification is analogous because the inverse staggered operator is antihermitian. The step must be done before the stochastic evaluation of the trace: once the random sources are introduced, cyclic invariance gets broken and will be restored only on average. Using Eq. (69) as a starting point, we obtain a drift which is already in the algebra (no need of taking the real part) and reads

70a $$\begin{aligned} F^{f}_\mu (x)_{ij}&=\frac{N_f}{N_c}\frac{\tau }{\beta }{{\mathrm{\Pi _\mathfrak {g}}}}\left( \sum _\beta \varphi ^{(\mu )}(x)_\beta \,\xi (x)^\dag _\beta \right) _{ij}&\qquad&\text {(Wilson fermions)} \end{aligned}$$

70b $$\begin{aligned} F^{f}_\mu (x)_{ij}&=\frac{N_f}{4N_c}\frac{\tau }{\beta }{{\mathrm{\Pi _\mathfrak {g}}}}\left( \varphi ^{(\mu )}(x)\,\xi (x)^\dag \right) _{ij}&\qquad&\text {(staggered fermions)}\,. \end{aligned}$$

In a similar fashion, it could be possible to show that also

71a $$\begin{aligned} F^{f}_\mu (x)_{ij}&=\frac{N_f}{N_c}\frac{\tau }{\beta }{{\mathrm{\Pi _\mathfrak {g}}}}\left( \sum _\beta {\tilde{\varphi }}^{(\mu )}(x)_\beta \,\psi (x)^\dag _\beta \right) _{ij}&\qquad&\text {(Wilson fermions)} \end{aligned}$$

71b $$\begin{aligned} F^{f}_\mu (x)_{ij}&=\frac{N_f}{4N_c}\frac{\tau }{\beta }{{\mathrm{\Pi _\mathfrak {g}}}}\left( {\tilde{\varphi }}^{(\mu )}(x)\,\psi (x)^\dag \right) _{ij}&\qquad&\text {(staggered fermions)} \end{aligned}$$

are legitimate expressions for the drift. All these new formulae are numerically different from those in Eqs. (29) and (33) but lead to the same results on average; clearly the advantage is that only half of the Lie derivative has to be computed.

## Fourier transforms with twisted boundary conditions

If *f*(*x*) is a periodic function defined on the $L^4$ lattice, its Fourier transform and inverse are

72 $$\begin{aligned} f(x) = \frac{1}{L^4}\sum _{p_\parallel } e^{ip_\parallel x}\tilde{f}(p_\parallel )\,, \qquad {\tilde{f}}(p_\parallel ) = \sum _x e^{-ip_\parallel x} f(x)\,, \end{aligned}$$

where $p_\parallel$ is the quantised vector $p_\parallel =\frac{2\pi }{L}(n_1,n_2,n_3,n_4)$ and the sum is to be read $\sum _{p_\parallel } = \sum _{n_1,n_2,n_3,n_4=0}^{L-1}$. Antiperiodicity in the direction ${\hat{\nu }}$ would lead again to Eq. (72) but with a quantised momentum $(p_\parallel )_\mu =\frac{2\pi }{L}n_\mu +\frac{\pi }{L}\delta _{\mu \nu }$.

**Twisted boundary conditions on a plane** Let us consider some $N_c\times N_c$ matrix *M*(*x*) (which for example can be a gauge link or the vector potential seen as matrices in colour space, or a fundamental fermion field seen as a matrix in colour-smell). We impose twisted boundary condition in the ${\hat{1}},{\hat{2}}$ plane so that

73 $$\begin{aligned} M(x+L\hat{1})= & {} \Omega _1 M(x)\Omega _1^\dag \,,\qquad \nonumber \\ M(x+L\hat{2})= & {} \Omega _2 M(x)\Omega _2^\dag \,, \end{aligned}$$

with $\Omega _2\Omega _1=z\Omega _1\Omega _2$, $z=z_{12}\in Z_N$. If we had just (anti)periodic boundary conditions, we would treat the matrix as $N_c^2$ independent scalar functions; twisted boundary conditions actually couple the different components, therefore in order to expand *M*(*x*) in plane waves we need to find a good basis for the matrix space: it can be proved (see Refs. [32, 33]) that the Fourier transform and its inverse are

74 $$\begin{aligned} M(x)= & {} \frac{1}{N_cL^4}\sum _{p_\parallel ,p_\perp } e^{ipx}\,\Gamma _{p_\perp } {\tilde{M}}(p_\parallel )_{p_\perp }\,,\qquad \nonumber \\ {\tilde{M}}(p_\parallel )_{p_\perp }= & {} \sum _x e^{-ip x}\,{{\mathrm{\mathrm {Tr}}}}\Gamma _{p_\perp }^\dag M(x)\,, \end{aligned}$$

where $p=p_\parallel +p_\perp$, $p_\perp$ is the quantised vector $p_\perp =\frac{2\pi }{N_cL}({\tilde{n}}_1,{\tilde{n}}_2,0,0)$ and the sum is to be read $\sum _{p_\perp } = \sum _{{\tilde{n}}_1,\tilde{n}_2=0}^{N_c-1}$. The matrices $\Gamma _{p_\perp }$ form the sought basis in the matrix space: assuming a twist with $z=\exp (2\pi i/N_c )$, we can choose for example

75 $$\begin{aligned} \Gamma _{p_\perp }=\Omega _1^{{\tilde{n}}_2}\Omega _2^{-{\tilde{n}}_1}\,. \end{aligned}$$

A different choice for *z* would have somehow reshuffled the exponents in Eq. (75). We see that the Fourier transform of *M*(*x*) is a scalar function $\tilde{M}(p_\parallel )_{p_\perp }$, but momentum has a finer resolution compared to (anti)periodic boundary conditions: spatial and colour degrees of freedom mix in momentum space. Moreover, traceless matrices naturally do not have a zero momentum component, because ${\tilde{M}}(p_\parallel )_0=\sum _x e^{-ip_\parallel x}{{\mathrm{\mathrm {Tr}}}}M(x)$.

**Twisted boundary conditions in three directions** The conditions in Eq. (73) are supplemented by

76 $$\begin{aligned} M(x+L\hat{3})=\Omega _3 M(x)\Omega _3^\dag \,, \end{aligned}$$

with $\Omega _3=\Omega _1^\rho \Omega _2^\sigma$ and $\rho ,\sigma$ span all the possible twist choices. It can be shown that Eq. (74) still holds but with a fine momentum $p_\perp =\frac{2\pi }{N_cL}({\tilde{n}}_1,{\tilde{n}}_2,\tilde{n}_3,0)$. The component ${\tilde{n}}_3$ is not a new degree of freedom but depends on the values of ${\tilde{n}}_1,{\tilde{n}}_2$. For example, in the case $z=\exp (2\pi i/N_c ),\rho =\sigma =1$, then $\tilde{n}_3=({\tilde{n}}_1+{\tilde{n}}_2)\mod N_c$. Other choices of $z,\rho ,\sigma$ just give a new relation between ${\tilde{n}}_3$ and $z,{\tilde{n}}_1,{\tilde{n}}_2$.

**Numerical implementation** The Fast Fourier Transform (FFT) algorithm encodes Eq. (72), $\text {FFT}[f(x)]={\tilde{f}}(p)$. We cannot apply directly the FFT to each matrix element of *M*(*x*), because the Fourier expansion has a dependence on $p_\perp x$. First, we need to project onto one of the $p_\perp$,

77 $$\begin{aligned} {\hat{M}}(x)_{p_\perp }=e^{-ip_\perp x}{{\mathrm{\mathrm {Tr}}}}\Gamma _{p_\perp }^\dag M(x)= \frac{1}{L^4} \sum _{p_\parallel } e^{ip_\parallel x} \tilde{M}(p_\parallel )_{p_\perp }\,, \end{aligned}$$

and then to each of these we apply the FFT,

78 $$\begin{aligned} {\tilde{M}}(p_\parallel )_{p_\perp }=\text {FFT}[{\hat{M}}(x)_{p_\perp }]\,. \end{aligned}$$

At the end, $N_c^2$ projections and $N_c^2$ FFTs have been performed. The inverse transform will be simply

79 $$\begin{aligned} {\hat{M}}(x)_{p_\perp }=\text {FFT}^{-1}[\tilde{M}(p_\parallel )_{p_\perp }]\, \end{aligned}$$

followed by

80 $$\begin{aligned} M(x) = \frac{1}{N_c} \sum _{p_\perp } e^{ip_\perp x}\, \Gamma _{p_\perp } {\hat{M}}(x)_{p_\perp }\,. \end{aligned}$$

Note that ${\tilde{M}}(p_\parallel )_{p_\perp }$ is a scalar function but the dependence on $p_\perp$ is through ${\tilde{n}}_1,{\tilde{n}}_2$, where each integer runs from 0 to $N_c-1$: this allows a representation of the Fourier transform again with a $N_c\times N_c$ matrix field, $\left( M(p_\parallel )\right) _{{\tilde{n}}_1{\tilde{n}}_2}$. Of course this has to be understood only as a useful representation of the momentum degrees of freedom, not as a matrix in colour space.

## Autocorrelations and cross-correlations

We consider a sample $\{a_i,b_i\}_{i=1}^N$ of measures of two observables *A*, *B* taken from the stochastic process at equilibrium. Let $\mathinner {\langle {A}\rangle }=a,\mathinner {\langle {B}\rangle }=b$ be the expectation values respectively of the observables *A*, *B*.The *cross-correlation function* is defined as

81 $$\begin{aligned} \Gamma _{AB}(t)=\mathinner {\langle {(a_i-a)(b_{i+t}-a)}\rangle }=\mathinner {\langle {a_ib_{i+t}}\rangle }-ab\,, \end{aligned}$$

where we used the fact that the expectation value is not dependent on *i* because the equilibrium distribution is time-independent. The cross-correlation function is not an even function, $\Gamma _{AB}(-t)=\Gamma _{BA}(t)$. In particular, $\Gamma _{AB}(0)=\text {Cov(A,B)}$ is the covariance between *A* and *B*. The average ${\bar{a}}=\frac{1}{N}\sum _{i=1}^Na_i$ is a stochastic variable that satisfies $\mathinner {\langle {{\bar{a}}}\rangle }=a$. The covariance between the estimators ${\bar{a}}$ and ${\bar{b}}$ is

82 $$\begin{aligned} \text {Cov}({\bar{a}},{\bar{b}})=&\mathinner {\langle {({\bar{a}}-a)(\bar{b}-b)}\rangle }=\frac{1}{N^2}\sum _{i,j=1}^N\Gamma _{AB}(i-j)\nonumber \\ =&\frac{\text {Cov(A,B)}}{N}\left[ 1+\sum _{r=1}^{N-1}\left( 1-\frac{r}{N}\right) \frac{\Gamma _{AB}(r)}{\Gamma _{AB}(0)}\right. \nonumber \\&\left. +\sum _{r=1}^{N-1}\left( 1-\frac{r}{N}\right) \frac{\Gamma _{AB}(-r)}{\Gamma _{AB}(0)}\right] \end{aligned}$$

but since the cross-correlation function is expected to drop exponentially at large times, it is possible to approximate

83 $$\begin{aligned} \text {Cov}({\bar{a}},\bar{b})\simeq \frac{\text {Cov(A,B)}}{N}\,(\tau _{AB}^\text {int}+\tau _{BA}^\text {int}) \end{aligned}$$

with the *integrated cross-correlation time*

84 $$\begin{aligned} \tau _{AB}^\text {int}=\frac{1}{2}+\sum _{r=1}^\infty \frac{\Gamma _{AB}(r)}{\Gamma _{AB}(0)}\,. \end{aligned}$$

We expect $\tau _{AB}^\text {int}\ne \frac{1}{2}$ when the observable *B* has some dependence on *A*. If *B* is independent of *A*, we can assume $\tau _{AB}^\text {int}=\frac{1}{2}$. An estimator for the cross-correlation function is

85 $$\begin{aligned} {\bar{\Gamma }}_{AB}(t)=\frac{1}{N-t}\sum _{i=1}^{N-t}(a_i-\bar{a})(b_{i+t}-{\bar{b}})\,. \end{aligned}$$

and the integrated cross-correlation time can be extracted in the Madras-Sokal approximation [64, 65]. Note that when $A=B$ then $\Gamma _{AA}(t)$ is the *autocorrelation function* and Eq. (83) becomes $\text {Var}(\bar{a})=2\tau _{AA}^\text {int}\text {Var}({\bar{a}})/N$, where $\tau _{AA}^\text {int}$ is the *integrated autocorrelation time*.

## Twisted lattice perturbation theory

Twisted lattice perturbation theory for the a pure gauge theory was introduced in Ref. [32] (see also Ref. [66]). Recently, the computation of Wilson loops has been treated in detail in Ref. [48]. Here we focus on two vertices, introducing Wilson and staggered fermions with smell in the fundamental representation. Feynman rules are fairly similar to those of lattice perturbation theory, apart from phases in propagators and vertices; all phases cancel in the first-order computations we considered. We recall also that the sum over momenta is inherited from the Fourier transform in Appendix C,

86

and each fermion loop has to be divided by $N_c$, i.e. by the numbers of smells running in the loop. The function $f(p_\perp ,p_\perp ^\prime )=z^{-{\tilde{n}}_1{\tilde{n}}_2^\prime }$ is introduced for convenience. The gluon propagator is

87 $$\begin{aligned} \mathinner {\langle {{\tilde{A}}_\mu (p)\tilde{A}_\nu (q)}\rangle }= & {} \delta _{p,q}\frac{(1-\delta _{p_\perp ,0})}{2}f(p_\perp ,p_\perp )\frac{1}{4\sum _{\rho }\sin ^2\left( \frac{p_\rho }{2}\right) }\nonumber \\&\quad \times \left[ \delta _{\mu \nu }-(1-\xi )\frac{\sin \left( \frac{p_\mu }{2}\right) \sin \left( \frac{p_\nu }{2}\right) }{\sum _{\sigma }\sin ^2\left( \frac{p_\sigma }{2}\right) }\right] \,,\nonumber \\ \end{aligned}$$

where $\xi$ is the gauge fixing parameter; note that the traceless property of the gauge field forces the propagator to vanish for $p_\perp =0$. The Wilson and staggered propagators are defined respectively in Eqs. (30) and (35). Below we write the fermion-fermion-gluon and fermion-fermion-gluon-gluon vertices in the Wilson and staggered case; $p_1, p_2$ are respectively the incoming and outgoing momenta of the fermions, $k_1, k_2$ are the outgoing momenta of the gluons.

**Wilson fermions**

88a $$\begin{aligned}&V_{ffg}(p_1,p_2,k_\perp )_{\mu }\nonumber \\&\quad =-g\,f(k_\perp ,p_{2\perp })\left[ i\gamma _\mu \cos \frac{1}{2}\left( p_1^A+p_2^A\right) _\mu \right. \nonumber \\&\qquad \left. +\sin \frac{1}{2}\left( p_1^A+p_2^A\right) _\mu \right] \end{aligned}$$

88b $$\begin{aligned}&V_{ffgg}(p_1,p_2,k_{1\perp },k_{2\perp })_{\mu \nu }\nonumber \\&\quad =-g^2\delta _{\mu \nu }f(k_{1\perp }+k_{2\perp },p_{2\perp })\,\frac{1}{2}[f(k_{1\perp },k_{2\perp })\nonumber \\&\qquad +f(k_{2\perp },k_{1\perp })]\,\nonumber \\&\qquad \times \,\left[ \cos \frac{1}{2}\left( p_1^A+p_2^A\right) _\mu -i\gamma _\mu \sin \frac{1}{2}\left( p_1^A+p_2^A\right) _\mu \right] \end{aligned}$$

**Staggered fermions** Here momentum conservation is made explicit, because the vertices are not diagonal in momentum space.

89a $$\begin{aligned}&V_{ffg}(p_1,p_2,k_1)_\mu \nonumber \\&\quad =-ig\,f(k_{1\perp },p_{2\perp })\,\cos \left( p_2+\frac{k_1}{2}\right) _\mu \,\nonumber \\&\qquad \times \,{\bar{\delta }}(-p_{1\parallel }+k_{1\parallel }+p_{2\parallel }+\pi {\bar{\mu }})\delta _{-p_{1\perp }+k_{1\perp }+p_{2\perp },0} \end{aligned}$$

89b $$\begin{aligned}&V_{ffgg}(p_1,p_2,k_1,k_2)_{\mu \nu }\nonumber \\&\quad =ig^2\,f(k_{1\perp }+k_{2\perp },p_{2\perp })\frac{1}{2}\left[ f(k_{1\perp },k_{2\perp })+f(k_{2\perp },k_{1\perp })\right] \,\nonumber \\&\qquad \times \,\sin \left( p_2+\frac{k_1}{2}+\frac{k_2}{2}\right) _\mu \,\nonumber \\&\qquad \times \,\delta _{\mu \nu }\,{\bar{\delta }}^{(4)}(-p_1+k_1+k_2+p_2+\pi {\bar{\mu }})\delta _{k_{1\perp }-p_{1\perp }+k_{2\perp }+p_{2\perp },0} \end{aligned}$$

## Code development for NSPT

We developed two independent NSPT codes in order to cross-check and improve our implementation.

PRlgt^10^ stems from the first NSPT codes developed by the Parma lattice gauge theory group, allowing for ${{\mathrm{{\mathrm {SU}}}}}(3)$ simulations with Wilson fermions. We implemented twisted boundary conditions, smell for Wilson fermions and added support for ${{\mathrm{{\mathrm {SU}}}}}(2)$ simulations. The code is tailored for perturbation theory. The underlying idea is to have base classes ptSU2 and ptSU3 that describe perturbative matrices. The operator * is overloaded with the Cauchy product, so that it is possible to write the product of two series in a natural way. This is one of the operations that, especially at high orders, becomes very time-consuming: thus, having perturbative matrices as base classes allows to keep the perturbative orders close in memory and to speed up the multiplication of series. In particular, the perturbative expansion is hardcoded to start from 1 for an element of the group and from 0 for an element of the algebra, in order to avoid multiplying by the identity or zero matrix; this choice also improves numerical stability in keeping the series within the group or algebra. All the other structures are built from the base classes by adding Lorentz, Dirac or lattice degrees of freedom. The fermion field too is described by matrices in colour-smell space. The update of the configuration is done one link at a time: this is possible, faster and less memory consuming for the first order integrator we are using; indeed the staples around a link can be computed also if the neighbour links have already been updated, since the effect of doing so gives higher-order effects in the time step. Twisted boundary conditions are implemented ad hoc for the Wilson action, as shown in Fig. 15: a system of twisted copies of the links on the boundary is updated at each Langevin step. The code makes heavy use of multithreading in all loops over lattice sites. Even though the performance of PRlgt is extremely good for small lattices, it is hard to scale to large volumes due to the scalar nature of the code.

We have also developed the GridNSPT code,^11^ based on the Grid library [68]. GridNSPT has been debugged against PRlgt, and we are able to obtain the very same outputs from these two completely different implementations (but staggered fermions have been implemented in GridNSPT only). The Grid library provides an environment where message passing, multithreading and vector parallelism are fully exploited: the lattice is geometrically decomposed into MPI domain, each one mapped to a set of processors; it is also overdecomposed over virtual nodes in order to fill a SIMD vector, assuring very high vectorisation efficiency. For example, on KNL and Skylake machines we can exploit the AVX-512 instruction set and a SIMD vector has room for 4 complex numbers in double precision; the virtual node decomposition results in the layout 1.1.2.2, where we are referring respectively to the coordinates *x*.*y*.*z*.*t*. Within the MPI task, multithreading is automatic because it is included in the closure of Grid lattice object expression templates. Grid incorporates $\hbox {C}++11$ internal template classes representing scalars, vector or matrices. We introduced a new template class representing a perturbative series, that embeds the overloading of the * operator.

All the structures are tensors built from these templates: for example, the gauge field is $\texttt {Lattice<iVector<iScalar}$ $\texttt {<iPert<iMatrix<vComplexD,Nc>,Np>{>},Nd>{>}}$, where (starting from the outer template) we have the lattice, Lorentz, spin, perturbative, colour structure and the base type is a vectorised complex number in double precision. With this in place, every operation in Grid is performed consistently with almost no modification. We rely on the Grid library for the optimal implementation of the gauge action and for the Wilson and staggered fermion kernel. Twisted boundary conditions have been implemented modifying the covariant circular shifts. Even though GridNSPT lacks of many optimisations compared to PRlgt (for example the Langevin update is not performed one link at a time, but all operations and shifts are performed on the lattice as whole), it allows to have a more flexible environment and to scale easily end very efficiently to large volumes.

## The nearest covariance matrix

If *C* is a covariance matrix, the corresponding correlation matrix is defined as

90 $$\begin{aligned} {\hat{C}}=S^{-1/2} \,C\, S^{-1/2}\,, \end{aligned}$$

where *S* is the matrix which is equal to *C* on the diagonal and vanishes everywhere else. ${\hat{C}}$ has 1 on the diagonal by construction; it might have some negative or zero eigenvalue if the estimator used in the determination of the covariance does not guarantee positive definiteness. Given ${\hat{C}}$, Higham’s algorithm [69] allows to find the nearest (in a weighted Frobenius norm) positive semidefinite matrix with unit diagonal. The core of the procedure is alternating a projection $P_S$ onto the space of positive semidefinite matrices and a projection $P_U$ onto the matrices with unit diagonal. The projection $P_S(X)=Y$ consists in

- diagonalising $X=U^T \,\Lambda \,U$, where *U* is an orthogonal matrix and $\Lambda$ is a diagonal matrix with the eigenvalues of *X* on the diagonal
- setting to zero all the negative elements in $\Lambda$, obtaining ${\tilde{\Lambda }}$
- returning $$Y=U^T \,{\tilde{\Lambda }} \,U$$.

The projection $P_U(X)$ consists simply in putting 1 on the diagonal of *X*. We refer to the original work for the presentation and proof of the complete algorithm: after some iterations, the algorithm converges and returns a matrix ${\hat{C}}_H$ which is positive semidefinite and has 1 on the diagonal.

However, the algorithm allows ${\hat{C}}_H$ to have some zero (within machine precision) eigenvalue, preventing the inversion of the covariance matrix. If this is the case, we additionally project ${\hat{C}}_H$ onto the space of positive definite matrices. This projection consists in

- diagonalising ${\hat{C}}_H=V^T \,\Gamma \,V$, where *V* is an orthogonal matrix and $\Gamma$ is a diagonal matrix with the eigenvalues of ${\hat{C}}_H$ on the diagonal
- identifying $\epsilon =\delta \lambda _{max}$, where $\lambda _{max}$ is the maximum eigenvalue and $\delta$ is the tolerance of the projection
- setting to $\epsilon$ all the diagonal elements of $\Gamma$ whose absolute value is smaller than $\epsilon$, obtaining ${\tilde{\Gamma }}$
- returning $${\hat{C}}_P=V^T \,{\tilde{\Gamma }} \,V$$.

In conclusion, the nearest covariance matrix is

91 $$\begin{aligned} C_P=S^{1/2} \,{\hat{C}}_P\, S^{1/2}\,. \end{aligned}$$

## Footnotes

1 One should note that one of the reason why the renormalon growth was correctly reproduced and the OPE correctly reconstructed is the adoption of twisted boundary conditions: in this way zero modes are absent and the theoretical picture is clear.

2 For convenience, we summarise our group theory conventions in Appendix A.

3 Obviously $\xi$ does not have any Dirac structure in the staggered case. The noise can be built from the independent generation of real and imaginary part with zero mean and variance 1 / 2.

4 Expanding in *ap* and sending the lattice size to infinity are operations that do not commute; in particular this gives rise to different series in the finite- and infinite-volume cases.

5 The different subsets are built by varying the number of initial configurations that are excluded in the analysis and by rejecting data at different rates.

6 We are grateful to M. García Pérez and A. González-Arroyo for providing us the gluon contribution in finite volume.

7 We mention that, in a theory with fermions, the operator $O_G$ must be combined with $m{\bar{\psi }}\psi$ to give a renormalisation group invariant quantity; moreover mixing with the operators $m{\bar{\psi }}\psi$ and  should also be considered [51, 52]. Clearly such complications are not present in the massless case and the operator  can be neglected in the following discussions since it vanishes when the equation of motion are used.

8 It is useful to keep in mind that other definitions of the gluon condensate are possible, see e.g. Ref. [54].

9 Our definition of the ambiguity differs from the one in Ref. [16] by a factor $\sqrt{\pi /2}$.
